# Rhizospheric *Bacillus* isolates control Fusarium wilt on cotton and enhance plant biomass and root development

**DOI:** 10.3389/fmicb.2025.1580937

**Published:** 2025-05-02

**Authors:** Seema Aslam, Muhammad Baber, Tahir Naqqash, Muhammad Javed, Sandra Bredenbruch, Florian M. W. Grundler, A. Sylvia S. Schleker

**Affiliations:** ^1^Institute of Molecular Biology and Biotechnology, Bahauddin Zakariya University, Multan, Pakistan; ^2^INRES, Department of Molecular Phytomedicine, Rheinische Friedrich-Wilhelms-University of Bonn, Bonn, Germany; ^3^All Pakistan Textile Mills Association, Islamabad, Pakistan

**Keywords:** antifungal activity, hydrolytic enzymes, siderophore, plant growth-promoting rhizobacteria, *Bacillus*

## Abstract

Cotton is a globally significant crop, serving as a source of natural fiber for the textile industry and contributing to various other products. Its economic importance is substantial, impacting livelihoods and international trade. However, cotton production faces numerous challenges, including Fusarium wilt caused by *Fusarium oxysporum* f. sp. *vasinfectum* (Fov), which can lead to significant yield and fiber quality losses. Plants alter their root exudate profiles in response to pathogens, often selectively enriching for beneficial rhizobacteria with antagonistic activity and plant growth-promoting traits. This study thus aims to characterize bacteria isolated from the rhizosphere of diseased cotton plants. The antifungal activity of 43 isolates was assessed against Fov *in vitro*. Eight of these inhibited Fov growth by 68.4 to 76.9%. 16S rRNA sequencing confirmed these isolates as *Bacillus* species. These eight *Bacillus* strains were further examined for their different modes of action *in vitro*, and their effect on cotton plants in greenhouse experiments challenged with Fov. All eight strains produced chitinases and pectinases, seven demonstrated cellulase and three protease activity, six produced urease, and five siderophores. Only *B. subtilis* SC11 exhibited phosphate solubilization activity. Seed treatments revealed that *B. subtilis* SC10 and *B. subtilis* SC11 were the standout treatments reducing Fov-caused symptoms by ~83% compared to Fov-inoculated control plants and most significantly improved plant growth and antioxidant activity. In detail, *B. subtilis* SC11 increased shoot and root dry weight by 160 and 250%, respectively. *B. subtilis* SC10 increased peroxidase activity by ~143% and ascorbate peroxidase activity by ~60%, while in *B. subtilis* SC11 treated plants superoxide dismutase activity increased by ~100%. *Bacillus* treatments effectively mitigated lipid peroxidation, achieving up to 91.4% reduction (*B. subtilis* SC10, *B. halotolerans* SC15), and decreased H₂O₂ accumulation by up to 58.4% (*B. halotolerans* SC32) compared to the Fov control. Principle component analysis revealed that regarding plant growth parameters, the treatments, and controls were distributed differentially across PC1 and PC2, with 60.30 and 15.62% data variance, respectively, showing the effectiveness of *Bacillus* isolates in greenhouse experiments. The findings of this study will contribute to the development of sustainable biocontrol strategies for managing Fusarium wilt in cotton.

## 1 Introduction

Pests and diseases lead to significant agricultural losses globally equaling about 550 billion dollars each year. Of these losses, 60% are due to pests, weather conditions, and weeds, while plant diseases, particularly those caused by fungal pathogens, are responsible for the remaining 40% (Etesami et al., 2023). Fungi alone are culpable for the destruction of over 125 million tons of key crops annually, including cotton wheat, maize, rice, potatoes, and soybeans (Shukla et al., 2022). Over the past decade, Pakistan’s cotton production has sharply declined from 14.81 million bales (one bale equals about 170 kg) in 2012–13 to just 5.5 million bales in 2022–24, causing an annual direct loss of approximately $4 billion and at least $15 billion in GDP. This drastic reduction is primarily attributed to climate change and the devastating impact of Fusarium wilt outbreaks during the monsoon season, leading to widespread crop failure. The persistent decline in cotton, often referred to as “white gold,” has severely affected the country’s textile industry and overall economic stability. “We thank M. Javed (personal communication, March 2025) for noting this ambiguity.” Current strategies for managing phytopathogens typically involve the use of synthetic pesticides. The potential detrimental environmental consequences of such agrochemicals, which may include biodiversity loss and pollution through toxicity and bioaccumulation, underscore the urgency to develop more sustainable alternative biocontrol methods, that utilize natural predators and antagonists of phytopathogens. These offer a promising alternative to chemical agents for protecting crops and ensuring plant health. Biological control agents especially plant growth-promoting rhizobacteria (PGPR) are becoming more prevalent in sustainable agriculture, displaying notable benefits for plant health across various regions (Landa et al., 2012).

Amongst PGPR the genera *Pseudomonas*, *Bacillus*, *Azoarcus*, *Klebsiella*, *Azospirillum*, *Enterobacter*, *Azotobacter, Serratia,* and *Rhizobium* are the most common and well-studied. They support plant growth through various mechanisms such as nitrogen fixation, solubilization of phosphate and other minerals, production of plant hormones, and suppression of pathogens through antagonistic actions. The effectiveness of PGPR can differ markedly due to variations in soil type, plant species, and availability of nutrients (Etesami and Adl, 2020). There is significant diversity within rhizobacteria species and genera; not all strains exhibit the same plant growth-promoting traits or abilities. The diverse range of characteristics and capabilities is owed to differences in the organisms’ genetic makeup and metabolic capacities. For practical agricultural use, PGPRs are processed into microbial formulations. These can be applied directly to seeds or in the soil to enhance the plant rhizosphere’s beneficial microbial population. When applied, PGPR can colonize plant roots and deliver direct nutrition to the plant or provide protection against soil-borne diseases (Abdelaziz et al., 2023). Using PGPR in crop production is considered a safer and more environmentally friendly alternative compared to synthetic chemicals. Their application leads to various plant growth improvements, such as increased seed germination rates, enhanced development of plant shoots and roots, and higher plant biomass, as well as to plant health improvements due to their antagonistic properties (Raklami et al., 2019). PGPRs can suppress phytopathogenic soil microbes through competition for nutrients and space, antibiotics, siderophore that sequester iron and make it less available to the phytopathogens, enzymes that degrade the cell walls of fungi, and induction of plant defense responses including systemic resistance (Nadeem et al., 2014).

*Bacillus* spp. stand out as exceptionally effective PGPR due to their multifaceted roles in promoting plant health and resilience. Their versatility stems from their ability of enhancing nutrient availability, serving as biocontrol agents by suppressing diseases, and improving overall soil health through various mechanisms. They exhibit diverse modes of action, including nitrogen fixation, phosphate solubilization, production of plant hormones, induction of systemic resistance, and synthesis of antimicrobial compounds. Furthermore, their capacity to withstand environmental stresses and form spores ensures their survival and consistent performance in diverse agricultural settings. The capacity of PGPR strains to colonize the plant root is also paramount. These combined attributes make *Bacillus* spp. invaluable tools for sustainable agriculture, offering a holistic approach to crop production while minimizing reliance on synthetic chemicals (Fira et al., 2018). Many strains of *Bacillus* spp. are well-known PGPR with strong antagonistic activity against *F. oxysporum*. This fungus is a member of the Ascomycota phylum, has a wide host range and is responsible for a variety of plant diseases including vascular wilts, root rots, head blights, and patch diseases (Liu and Zhang, 2021). It attacks plants by infiltrating mainly seedling roots, facilitated by wounds, before colonizing the vascular system of the host. This pathogen leads to substantial crop losses in many economically valuable plant species, such as cotton, cereals, potatoes, tomatoes, ornamental flowers, date palms, oil palms, and bananas (Zaim et al., 2016).

PGPR use for controlling Fusarium wilt in cotton holds great promise. Additionally, integrating PGPR usage with other crop management practices such as the use of disease-resistant varieties, crop rotation, and optimal fertilization can lead to a more robust integrated disease management system (Fu et al., 2017). Hence, promoting the use of antagonistic PGPR can be an essential part of a strategy for maintaining the health of cotton crops and ensuring high yields, thereby supporting the livelihoods of cotton farmers and the textile industry at large.

Further research is crucial to explore the effects of antagonistic PGPR on cotton crop development and their capacity for endurance under fungal stress. So far, the microbiome of the cotton rhizosphere from continuous cotton-wheat rotation systems was not specifically explored in this respect. Therefore, our research aims to investigate PGPR isolated from the cotton rhizosphere of continuous cotton-wheat rotation fields and evaluate their antagonistic effect against the phytopathogenic fungus *Fusarium oxysporum* f. sp. *vasinfectum* (Fov) both *in vitro* and in the greenhouse as well as the isolates’ plant growth promoting properties. This approach is significant because PGPR strains isolated from this specific environment are more likely to be adapted to the local soil conditions and exhibit enhanced biocontrol potential against Fov in this particular cropping system. This research can provide valuable insights into developing tailored biocontrol strategies for cotton-wheat rotations. Fov was selected for this study due to its prevalence worldwide, including the United States, India, China, Pakistan, and Africa as well as the limited efficacy of current control strategies, which cause substantial economic losses in major cotton-producing regions worldwide.

## 2 Materials and methods

### 2.1 Bacterial isolates

Rhizobacteria used in this study were isolated, purified, and preliminary screened for their plant growth-promoting and biocontrol traits at the Institute of Molecular Biology and Biotechnology, Bahauddin Zakariya University, Multan, Pakistan (Aslam et al. unpublished data). Fields infected with Fov were selected for rhizobacteria sampling. Cotton plants exhibiting relatively mild Fov disease symptoms were chosen based on the hypothesis that Fov infection alters root exudate profiles, potentially enriching for rhizobacteria with antagonistic activity against Fov. Rhizosphere soil tightly adhered to the roots was collected, and bacterial isolates were obtained using serial dilution plating (Juhnke et al., 1987). 43 rhizobacteria isolated from five distinct cotton rhizosphere samples were subsequently tested for their *in vitro* antifungal activity against Fov. Further biochemical assays for best performing isolates were performed to elucidate the biocontrol capabilities and plant growth-promoting properties of these rhizobacteria.

### 2.2 *In vitro* antifungal assay

*Fusarium oxysporum* f. sp. *vasinfectum* (Fov) was obtained from the National Institute for Biotechnology and Genetic Engineering (NIBGE), Faisalabad, Pakistan, where it was originally isolated from the rhizosphere of cotton. *Fusarium graminearum* (CSB 5–15), *Leptosphaeria maculans* (DSM 62910) and *Cercospora beticola* (DSM 621607) were obtained from INRES-Molecular Phytomedicine University of Bonn, Germany. Each fungal culture was taken from a glycerol stock (20%) and inoculated on potato-dextrose agar (PDA) in the Petri dishes (20 g dextrose, 15 g agar, and 4 g potato starch in 1 L dH_2_O) for further use in dual culture assays. All 43 isolates from relevant glycerol stocks were streaked on LB agar (5 g yeast extract, 5 g NaCl, 10 g trypton, and 15 g agar-agar in 1 L dH_2_O) for further use. All 43 isolated rhizobacteria were evaluated for *in vitro* antifungal activity by performing a dual-culture assay on PDA in Petri dishes against Fov (Sakthivel and Gnanamanickam, 1986). The eight best performing isolates were then used to check antifungal activity against *F. graminearum*, *L. maculans* and *C. beticola* in order to get an idea on their specificity. Biocontrol agents exhibiting broad-spectrum antimicrobial activity are generally more advantageous for field applications compared to agents with activity limited to one or a few pathogens. This broader efficacy can offer more robust and comprehensive disease control in complex field environments where multiple pathogens may be present (Ali et al., 2014). A fungal (≈5 mm^2^) plug was placed at the center of the plates, followed by streaking of freshly grown bacterial isolates 3 cm away from fungal plug. The plates were then incubated at 28 ± 2°C for 5 to 7 days in the dark. In addition, a control was set up by inoculating fungus onto the center of a Petri dish under the same incubation conditions but without bacterial treatment. Three biological replicates each with three technical replicates were performed. To determine antifungal activity, the following formula was used.


$$\mathrm{Fungal growth inhibition (\%)=}\left[1-\left(\frac{{\mathrm{Fungal growth}}_{\mathrm{Treated variant}}}{{\mathrm{Fungal growth}}_{\mathrm{Control}}}\right)\right]\times 100$$

### 2.3 Identification of the most potent rhizobacteria isolates

Based on the observed antifungal performance of the 43 rhizobacteria, 8 isolates designated as SC5, SC10, SC11, SC15, SC30, SC32, SC41, and SC42 were selected and subjected to 16S rRNA gene amplicon sequencing. Therefore, the isolates were cultured on LB agar plates and incubated at 28 ± 2°C for 24 h in the dark. An individual colony was transferred into 100 μL of sterile nuclease-free H_2_O, incubated for 20 min at 99°C and then centrifuged (4000xg) for 2 min. Subsequently, the supernatant containing bacterial DNA was used in a PCR with the universal primer pair fD1 (AGAGTTTGATCCTGGCTCAG) and rD1 (AAGGAGGTGATCCAGC) (Weisburg et al., 1991). Fifty microliters of the total reaction mixture were prepared using DreamTaq PCR Master Mix (Thermo Fisher Scientific) (24 μL), nuclease-free water (16 μL), 10 μM forward primer (2 μL), 10 μM reverse primer (2 μL), and bacterial DNA (6 μL). PCR cycling conditions were 95°C for 5 min, followed by 35 cycles at 95°C for 1 min, 56°C for 30 s, 72°C for 1 min, and final extension at 72°C for 10 min. The PCR product was run on a 1% TAE-agarose gel for 50 min at a constant 80 V. Subsequently, the required DNA fragments of ^~^1.5 kbp were cut and transferred into autoclaved Eppendorf tubes. DNA was purified from the gel by using a DNA purification kit (Thermo Fisher Scientific) according to the manufacturer’s instructions and sent to Eurofins Genomics (Germany) for sequencing. NCBI BLAST was used to analyze the obtained sequences by comparison to already deposited sequences in the Gene Bank database. After the initial blast against the whole NCBI database to get an idea of the identity of the isolates, the blast analysis was done with all type strains of a particular group to exclude incorrectly annotated entries (including several type strains utilized in the analysis and the selected group). ClustalW in Molecular Evolutionary Genetics Analysis Version 11 (MEGA 11) software was used to align highly homologous sequences and generate neighbor-joining trees. Phylogenetic tree nodes were statistically supported by bootstrap replication with 1000 replicates using MEGA 11 (Ki et al., 2009).

### 2.4 Extracellular enzymatic activities

The eight isolates of rhizobacteria (SC5, SC10, SC11, SC15, SC30, SC32, SC41, and SC42) that performed high antifungal activity were investigated for the production of chitinase, cellulase, pectinase, protease, and urease activities. All assays to identify extracellular enzymatic activities represented a qualitative approach. To determine chitinase, cellulase, and pectinase activities, the bacteria were inoculated on mineral salt (MS) agar media (1 g KH_2_ PO_4_, 5 g NaNO_3_, 2 g K_2_HPO_4_, 0.1 g CaCl_2_, 0.1 g KCl, 0.5 g MgSO_4_, 7H_2_ O, 0.02 g FeSO_4_, 7H_2_ O, 15 g agar in 1 L dH_2_O) supplemented with 1% colloidal chitin [prepared by treating chitin from crab shells with HCl following the protocol described by Li et al. (2019)] 1% carboxy-methylcellulose (CMC), and 1% pectin, respectively, (Chaiharn et al., 2019). A negative control without bacterial inoculation on supplemented MS agar was also used. After inoculation of a single pure colony of the respective bacterial isolate, plates were incubated at 28 ± 2°C for 48 h. Then, for better visualization plates were immersed in a Lugol solution comprising potassium iodide (KI;1% w/v) and Iodine (I_2_;0.5% w/v) in dH_2_O. Skim milk medium (10 g skim milk powder, 5 g tryptone, 2.5 g yeast extract, 1 g dextrose, and 15 g agar in 1 L dH_2_O) was used for the determination of protease activity. The formation of a zone of clearance around bacterial colonies indicated extracellular enzymatic activities (O'Sullivan et al., 1991).

For the determination of urease activity, urea agar base supplemented with 40% urea was used. The change in color from yellow to pink represented urea hydrolysis (Goswami et al., 2015). Three biological replicates each with three technical replicates were performed.

### 2.5 Siderophore production

#### 2.5.1 Qualitative assay

Siderophore secretion was qualitatively determined by using an LB agar medium containing Chrome Azurol S (CAS) as an indicator dye, Fe^3+^ solution, and hexadecyl trimethyl ammonium bromide (HDTMA). A single pure colony of the respective rhizobacteria isolate was inoculated onto a CAS agar plate and incubated for 72 h at 28 ± 2°C. The appearance of an orange-yellow zone around the bacterial colony represents the production of siderophore (Schwyn and Neilands, 1987). This experiment was performed in three biological replicates each with three technical replicates.

#### 2.5.2 Quantitative assay

For siderophore quantification, a single pure colony of each rhizobacteria from overnight bacteria culture was inoculated in 100 mL LB broth in a 500 mL conical flask and after 72 h of shaking (150 rpm) incubation at 28 ± 2°C supernatant was collected followed by centrifugation (9,000xg, 5 min). 100 μL supernatant of each bacterial culture was transferred into a separate well of a 96-well microtiter plate followed by the addition of an equal volume of CAS reagent. The optical density of each sample was recorded at 630 nm followed by incubation (Arora and Verma, 2017). Three biological and four technical replicates were used and siderophore production was calculated in percent siderophore unit (psu) (Schwyn and Neilands, 1987).

### 2.6 P-solubilization

A qualitative assay for P-solubilization activity of rhizobacteria was performed by using Pikovskaya’s agar media following the protocol described by Pikovskaya (1948). Fresh bacteria culture was inoculated in the center of Pikovskaya’s agar plates. Formation of the zone of clearance around the bacterial growth after 3 to 5 days of incubation at 28 ± 2°C represented the P-solubilization activity of bacteria. Three biological replicates each with three technical replicates were performed.

### 2.7 Indole 3-acetic acid production

To determine indole-3-acetic acid (IAA) production by rhizobacteria, a single colony of each isolate was inoculated into 50 mL of LB broth media both without and amended with tryptophan (100 mg /L) in 250 mL conical flasks and incubated at 28 ± 2°C for 72 h. After incubation, the cultures were centrifuged, and 150 μL of supernatant was mixed with an equal volume of Salkowski’s reagent in a 96 well plate. The cultures were incubated in the dark at 28 ± 2°C for 30 min. Development of a pink color indicates successful IAA production (Glickmann and Dessaux, 1995). Three biological replicates each with three technical replicates were performed.

### 2.8 Greenhouse experiment

#### 2.8.1 Experimental design

A pot experiment using a completely randomized design and eight technical and two biological replicates for each treatment was conducted to evaluate the efficacy of the 8 best performing isolates against Fov in cotton plants in the greenhouse of the Institute of Molecular Biology and Biotechnology, Bahauddin Zakariya University, Multan. Soil for this experiment was collected from a nursery in Multan. The physicochemical analysis of the soil used in this experiment is given in Supplementary Table 1. Cotton seeds of the commercially available MNH-1020 (Fov susceptible) variety were used. MNH-1020 is a commercial cultivar developed by the Cotton Research Institute (CRI) Multan, Pakistan. Ten treatments were designed as illustrated below:


$$T1=\mathrm{No}\;\mathrm{treatment}–Fov\;\left(\mathrm{uninfected control}\right)$$

$$T2=\mathrm{No}\;\mathrm{treatment}+Fov\;\left(\mathrm{infected control}\right)$$

T3=SC5+Fov

T4=SC10+Fov

T5=SC11+Fov

T6=SC15+Fov

T7=SC30+Fov

T8=SC32+Fov

T9=SC41+Fov

T10=SC42+Fov

In all 10 treatments sterilized cotton seeds were used. One-week post sowing, plant thinning was performed so that only one healthy plant in each pot remained. Throughout the experiment, greenhouse condition was constantly maintained at a temperature ranging from 25 to 30°C, 16 h photoperiod, and 60% humidity.

#### 2.8.2 Bacterial inoculum

A pure colony of each of the 8 isolates (already growing on LB agar plate) was separately inoculated into 100 mL LB media in a conical flask (250 mL) and incubated for 48 h at 28 ± 2°C in a shaking incubator at 120 rpm. The cultures were centrifuged in 50 mL falcon tubes for 10 min at 4000xg, the supernatant was discarded and the pellet was suspended in sterile dH_2_O. 1 × 10^8^ CFU mL^−1^ of each isolate was used for cotton seed treatment (Eddin et al., 2007). In T3 to T10 sterilized cotton seeds were soaked in the respective isolate inoculum for 15 min. Then three seeds from each treatment were sown in individual pots in eight replicates. In the T1 and T2 only sterilized seeds were planted.

#### 2.8.3 Fungal inoculum

Fov (loop full culture) was inoculated in 700 mL of potato dextrose broth (PDB) (20 g dextrose, and 4 g potato starch in 1 L dH_2_O) in 1 L conical flask (four 1 L conical flasks each with 700 mL PDB) and incubated for 15 days at 28 ± 2° C in the shaking incubator at 120 rpm. After 15 days, PDB with growing fungus was centrifuged in a 50 mL falcon tube at 4000xg for 10 min to obtain fungus pellets. The fungal pellets were suspended in sterile dH_2_O to obtain a density of 3 × 10^7^ spores/ml (Dilfuza and Dilfuza, 2011). This fungal inoculum was injected into soil near plant roots to infect soil (3 × 10^7^ spores/kg of soil) of treatments T2 to T10 2 weeks post sowing. Treatment T1 (without bacterial and fungal inoculation) was used as a control in this experiment. T2 (only with fungal inoculation) was used as a control to evaluate the infection severity. The plants were properly irrigated and daily observed for symptoms of a fungal infection.

The disease severity of Fusarium wilt was calculated 45 days after Fov inoculation based on leaf yellowing and root discoloration on a scale of 0 to 5, representing increasing severity: 0 indicates no symptoms; 1 indicates 1–20% light brown vascular discoloration and yellowing leaves; 2 indicates 20–40% light brown vascular discoloration and yellowing leaves; 3 indicates 40–60% dark brown vascular discoloration and yellowing leaves; 4 indicates 60–80% dark brown vascular discoloration and yellowing leaves; and 5 indicates 80–100% yellowish leaves and dark brown or dead vascular systems. Disease severity was then calculated using the formula:


$$\mathrm{Disease severity}=\Sigma \;\left[\begin{array}{l} \left(N\;x\;0\right)+\left(N\;x\;1\right)+\left(N\;x\;2\right)+\\ \left(N\;x\;3\right)+\left(N\;x\;4\right)+\left(N\;x\;5\right) \end{array}\right]/5\;x\;T$$

where N = number of plants at each symptom grade 0 to 5, and T = total number of plants (multiplied by maximum symptom grade 5).

The percentage reduction in Fusarium wilt incidence was calculated using the following formula:


$$\mathrm{Reduction percentages}\;\left(\%\right)=\left(A-B\right)/A\times 100$$

where A represents the disease incidence (disease severity) of the positive control and B represents the disease incidence of the treatment (El-Sersawy et al., 2021).

#### 2.8.4 Determination of growth parameters

After 45 days post inoculation (DPI) of Fov cotton plants were uprooted, main root and shoot length as well as root and shoot fresh weight were determined after washing and carefully drying the roots with tissue paper. In order to obtain the dry weight, roots and shoots half of plants (*n* = 4) from each biological replicate were oven-dried for 2 weeks at 65°C before weighing. Half of plants (*n* = 4) from each biological replicate were preserved in liquid nitrogen and stored at – 80°C for the measurement of oxidative stress and antioxidants.

### 2.9 Measuring oxidative stress

#### 2.9.1 Lipid peroxidation

Malondialdehyde (MDA) content was measured to determine lipid peroxidation. Fifty-nine days old cotton plants (T1 to T10) as describes in 2.7.2, were uprooted at 45 DPI with Fov, except for T1, which served as a non-inoculated control. The plants were immediately preserved in liquid nitrogen at – 80°C for further analysis. Three leaf samples were collected separately from each plant. Five milliliters of 0.1% trichloroacetic acid (TCA) solution were used to homogenize 200 g of cotton leaves that were preserved in liquid nitrogen and the homogenized solution was centrifuged for 5 min at 10,000 rpm. One milliliter of supernatant was mixed with 3 mL of 5% TCA and 1% TBA (thiobarbituric acid) solution. After heating for 30 min in a water bath at 95°C the final mixture was centrifuged for 5 min at 5000 rpm or 30 min. The supernatant (300 μL) was collected in a 96 well plate to measure the absorbance of samples at 532 and 600 nm by using a spectrophotometer. The extinction coefficient value (155 μM^−1^ cm^−1^) was used to estimate the actual value of samples (Heath and Packer, 1968). This experiment was performed in two independent biological replicates each with 12 technical replicates per treatment.

#### 2.9.2 Hydrogen peroxide

To determine the H_2_O_2_, 0.2 grams of leaves from each treatment as described in 2.8.1 were grounded in 0.1% TCA (2 mL), followed by centrifugation at 10,000 rpm for 15 min. Subsequently, 500 μL of 10 mM potassium phosphate (KP) buffer (pH 7.0) and 1 mL of 1 M Potassium Iodide (KI) were added in 500 μL of supernatant. The optical density (OD) of the mixture was then measured at 390 nm (Velikova et al., 2000). This experiment was performed in two independent biological replicates each with 12 technical replicates per treatment.

### 2.10 Determination of antioxidant activity

#### 2.10.1 Preparation of enzyme extract from cotton leaves

Leaves samples as described in section 2.8.1 were collected. 50 mM KP buffer was used to homogenize cotton leaf samples for the estimation of enzymatic antioxidant activity. Pre-chilled pestle and mortars were used for grinding. The reaction mixture was centrifuged at 12,000 rpm for 20 min at 4°C. Supernatant was collected for antioxidant analysis.

#### 2.10.2 Catalase

Catalase (CAT) activity was assessed by monitoring the decomposition of H_2_O_2_ at 240 nm. The reaction mixture consisted of 1 mL of 50 mM KP buffer, 450 μL of 5.9 mM H_2_O_2,_ and 50 μL of enzyme extract. Spectrophotometer was used to measure the absorbance at 240 nm after the intervals of 20 s up to 2 min (Aebi, 1984). The experiment was performed in two independent biological replicates each with 12 technical replicates per treatment.

#### 2.10.3 Ascorbate peroxidase

For Ascorbate peroxidase (APX) reaction mixture consists of 207 μL of 50 mM KP buffer, 7.6 μL of 300 mM H_2_O_2_, 7.6 μL of ascorbic acid and 7.6 μL of enzyme extract. Absorbance was measured at 290 nm (Dhindsa and Matowe, 1981). This experiment was performed in two independent biological replicates each with 12 technical replicates per treatment.

#### 2.10.4 Peroxidase

Peroxidase (POD) activity was assessed according to the method outlined by Maehly (1954) with slight modifications. The reaction mixture was comprised of 50 mM KP buffer (pH 7.8), 50 μL of enzyme extract, and 0.2 mL of guaiacol (20 mM). 0.25 mL of H_2_O_2_ (40 mM) was added in the reaction mixture to initiate the reaction. Absorbance readings were taken at 470 nm using a spectrometer at 20-s intervals for 2 min. This experiment was performed in two independent biological replicates each with 12 technical replicates per treatment.

#### 2.10.5 Superoxide dismutase

The activity of superoxide dismutase (SOD) was measured using the nitro blue tetrazolium (NBT) photoreduction method developed by Dhindsa and Matowe (1981). The reaction mixture consisted of 100 μL of enzyme extract, 50 μL of 33 mM NBT, 50 μL of 0.003 mM riboflavin 100 μL of 10 mM L-methionine, and 250 μL of 50 mM KP buffer. This mixture was exposed to a 15 W lamp for 30 min and then the lamp was turned off to stop the reaction. Control experiments were conducted with illuminated and non-illuminated reactions without enzyme extract. The absorbance was measured at 560 nm. This experiment was performed in two independent biological replicates each with 12 technical replicates per treatment.

### 2.11 Statistical analysis

The data collected from pot experiments were statistically analyzed using the analysis of variance method, based on the completely randomized design layout and programmed in the software Statistix 8.1. Differences between the means of each treatment were tested using the least significant difference LSD test at a 5% level of significance. The data were also analyzed using principal component analysis (PCA) to determine the relationship between the growth parameters and treatment using XLSTAT (Garcia et al., 2019).

## 3 Results

### 3.1 Screening for antifungal activity

Out of 43 isolates 31 isolates performed antifungal activity by inhibiting Fov colony growth by 3.5 to 76.9% in dual culture plate assay (Supplementary Table 1, Supplementary Figure 1). Eight bacterial isolates exhibiting the highest antifungal activity against Fov, ranging from 66.7 to 76.9% inhibition, were evaluated for their broader antifungal activity against three additional fungal strains. These isolates demonstrated a wider range of inhibition against *F. graminearum*, *L. maculans*, and *C. beticola*, ranging from 62.9 to 93% (Table 1). The eight isolates were selected for molecular characterization and further experiments were performed for the analysis of their biocontrol and plant growth-promoting potential.

**Table 1:** *In vitro* antagonistic potential (% inhibition of fungal growth) of eight rhizobacteria against four fungal phytopathogens.

Isolates	*Fusarium oxysporum* f.sp. *vasinfectum* (% growth inhibition)	*Fusarium graminearum* (% growth inhibition)	*Leptosphaeria maculans* (% growth inhibition)	*Cercospora beticola* (% growth inhibition)
*B. stercoris* SC5	72.2 ± 6.3	68.7 ± 3.4	91.7 ± 1.9	84.4 ± 3.9
*B. subtilis* SC10	70.3 ± 3.5	67.2 ± 2.5	83.1 ± 6.2	68.1 ± 3.9
*B. subtilis* SC11	68.9 ± 3.7	79.2 ± 8.9	90.0 ± 2.5	76.5 ± 3.3
*B. halotolerans* SC15	75.9 ± 3.8	62.9 ± 6.1	88.3 ± 3.1	77.0 ± 5.5
*B. halotolerans* SC30	70.3 ± 4.7	73.1 ± 2.5	93.0 ± 1.0	79.2 ± 2.6
*B. halotolerans* SC32	76.9 ± 4.6	67.3 ± 11.2	92.1 ± 1.5	80.1 ± 4.9
*B. subtilis* SC41	68.5 ± 3.9	77.0 ± 5.9	87.3 ± 1.0	75.6 ± 2.5
*B. subtilis* SC42	66.7 ± 5.7	70.8 ± 5.7	91.7 ± 3.2	77.8 ± 1.3

A dual culture experiment was performed with three biological replicates each with three technical replicates.

### 3.2 16S rRNA identification of the most potent rhizobacteria isolates

**Figure 1:**
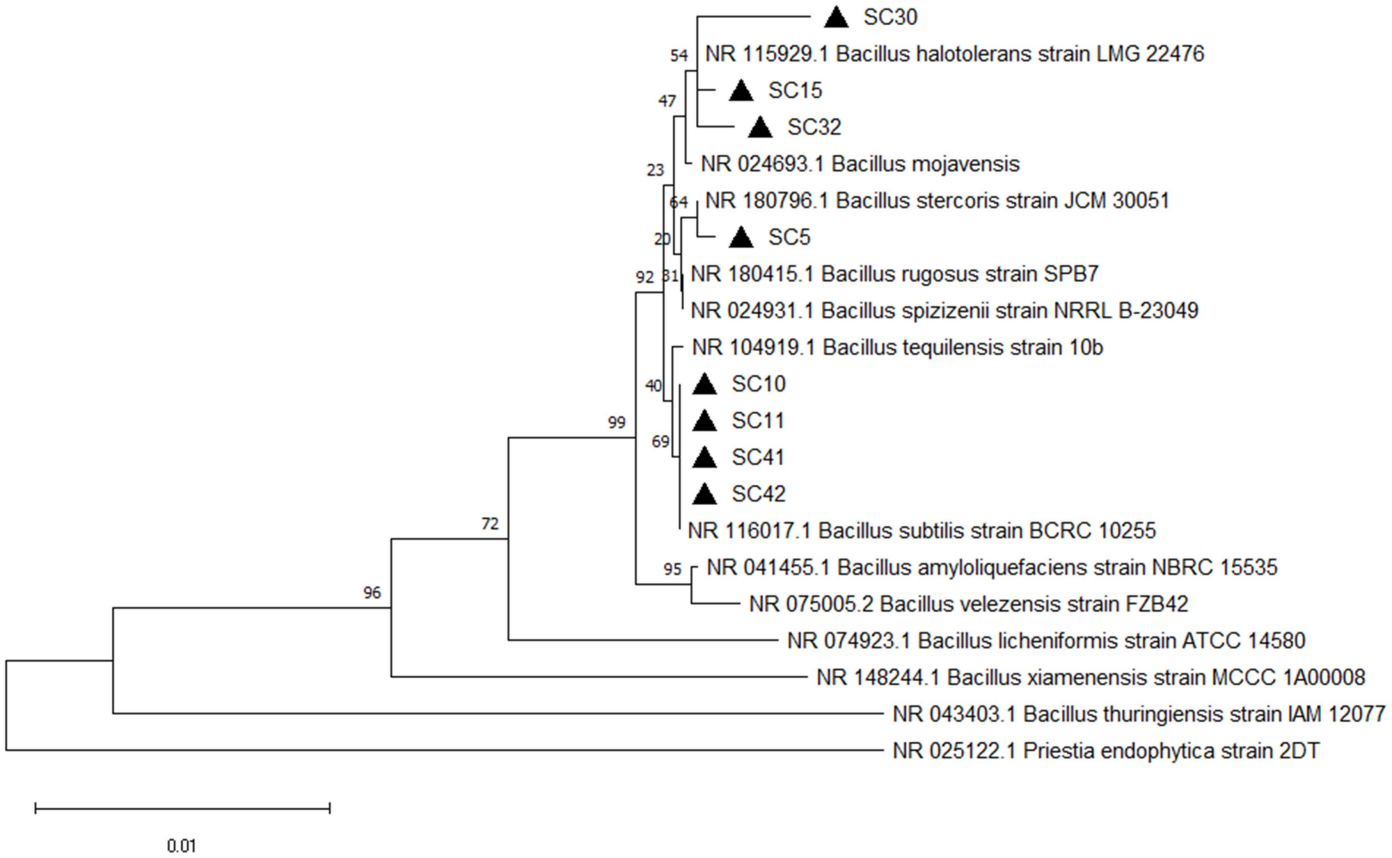
Phylogenetic tree of isolates *Bacillus stercoris* SC5, *Bacillus subtilis* (SC10, SC11, SC40, SC42), *Bacillus halotolerans* (SC15, SC30, SC32) and their closest relatives based on 16S rRNA sequence. The phylogenetic tree was constructed using the neighbor-joining (method in MEGA11 software). The bootstrap values are shown at the branch points.

The eight rhizobacteria isolates designated SC5, SC10, SC11, SC15, SC30, SC32, SC41, and SC42 that possessed high antifungal activity against 4 phytopathogenic fungi were identified on molecular level by 16S rRNA sequencing. Sequence analysis revealed that all eight isolates are a member of the genus *Bacillus*. SC5 was highly similar to *B. stercoris*. SC10, SC11 SC41, and SC4 were highly similar to *B. subtilis*. SC15, SC30, and SC32 were highly similar to *B. halotolerans* (Figure 1). The phylogenetic tree contains the type strains that are more closely related to the isolates under investigation. The sequences are available at GenBank under the accession numbers PV147758 to PV147765.

### 3.3 Extracellular enzymatic activities

**Figure 2:**
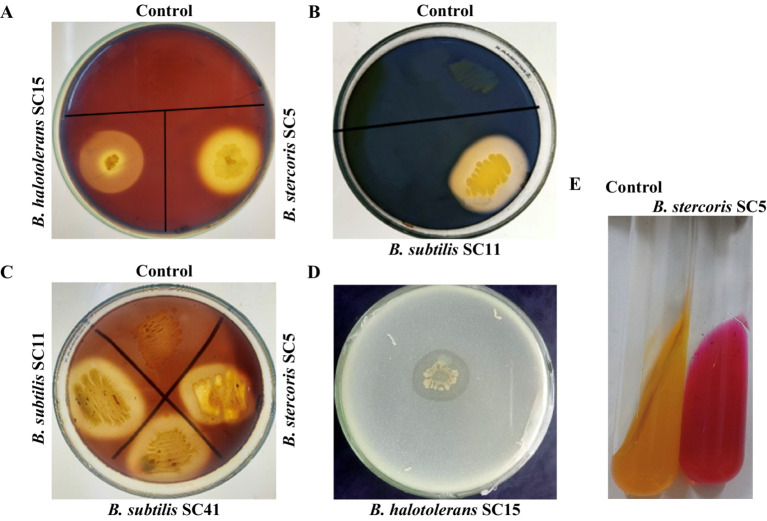
Plate assays for screening of enzymatic activities of antagonistic rhizobacteria. Representative examples. **(A)** Chitinase enzyme production on medium amended with 1% colloidal chitin indicated by the development of a halo zone around the bacterial colony. **(B)** Cellulase production on medium amended by 1% carboxymethyl cellulose (CMC) indicated the development of a halo zone around the bacterial colony. **(C)** Pectinase enzyme production on medium amended by 1% pectin indicated by the development of halo zone around bacterial colony. **(D)** Production of protease on skim milk medium indicated by development of halo zone around bacteria colony. **(E)** Urease production indicated a change in the color of the medium.

The production of extracellular enzymes is a key trait of many successful biocontrol agents, contributing significantly to their ability to suppress fungal pathogens and promote plant health. All isolates performed low to strong chitinase activity. *B. subtilis* isolates SC10, SC11, SC41, and SC42 performed low chitinase activity, *B. halotolerans* isolates SC15, SC30 and SC32 performed strong chitinase activity and *B. stercoris* SC5 performed very strong chitinase activity (Table 2, Figure 2A). *B. stercoris* SC5, *B. subtilis* isolates SC10, SC11, SC42, and *B. halotolerans* isolates SC15, SC30, SC32 performed strong pectinase activity and only *B. subtilis* SC41 performed low pectinase activity (Table 2, Figure 2B). All isolates but *B. halotolerans* SC30 performed cellulase activity, with *B. subtilis* isolates SC10, SC11, and *B. halotolerans* SC15 even performing very strong activity (Table 2, Figure 2C). The three isolates *B. stercoris* SC5, *B. halotolerans* SC15, and *B. halotolerans* SC30 performed strong protease activity (Table 2, Figure 2D). Enzymatic activities of all above mentioned enzymes were categorized based on the width of zone clearance. The four isolates *B. stercoris* SC5, *B. subtilis* isolates SC10, SC42, and *B. halotolerans* SC30 performed low, and the two isolates *B. subtilis* SC11 and *B. halotolerans* SC32 strong urease activity (Table 2). Urease activity was categorized based on the change in color of media from yellow to dark pink color (Figure 2E). All traits exhibited by these bacteria are summarized in Table 2.

**Table 2:** Biocontrol and plant growth promoting traits of bacterial isolates characterized based on their activity.

Isolates	Chitinase activity	Cellulase activity	Pectinase activity	Protease activity	Urease activity	Siderophore production (psu)	P-solubilization
*B. stercoris* SC5	**+++**	**++**	**++**	**++**	**+**	−	**−**
*B. subtilis* SC10	**+**	**+++**	**++**	**−**	**+**	31.1^B^ ± 1.3	**−**
*B. subtilis* SC11	**+**	**+++**	**++**	**−**	**++**	33.6^A^ ± 1.4	**+**
*B. halotolerans* SC15	**++**	**+++**	**++**	**++**	**−**	30.6^B^ ± 1.6	**−**
*B. halotolerans* SC30	**++**	**−**	**++**	**++**	**+**	**−**	**−**
*B. halotolerans* SC32	**++**	**+**	**++**	**−**	**++**	**−**	**−**
*B. subtilis* SC41	**+**	**++**	**+**	**−**	**−**	18.9^C^ ± 1.2	**−**
*B. subtilis* SC42	**+**	**++**	**++**	**−**	**+**	17.4^C^ ± 1.1	**−**

+++ (with 2.1 cm to 2.5 cm zone width) very strong activity; ++ (1.1 cm to 2 cm zone width) strong activity; + (up to 1 cm zone width) low activity; − no activity. All qualitative experiments were performed in three biological replicates each with three technical replicates. Siderophore was quantified by using four technical and three biological replicates. Values represent the average + standard deviation of three biological replicates. Significant differences between treatments are indicated by different letters.

### 3.4 Siderophore production, P-solubilization and indole 3-acetic acid production

**Figure 3:**
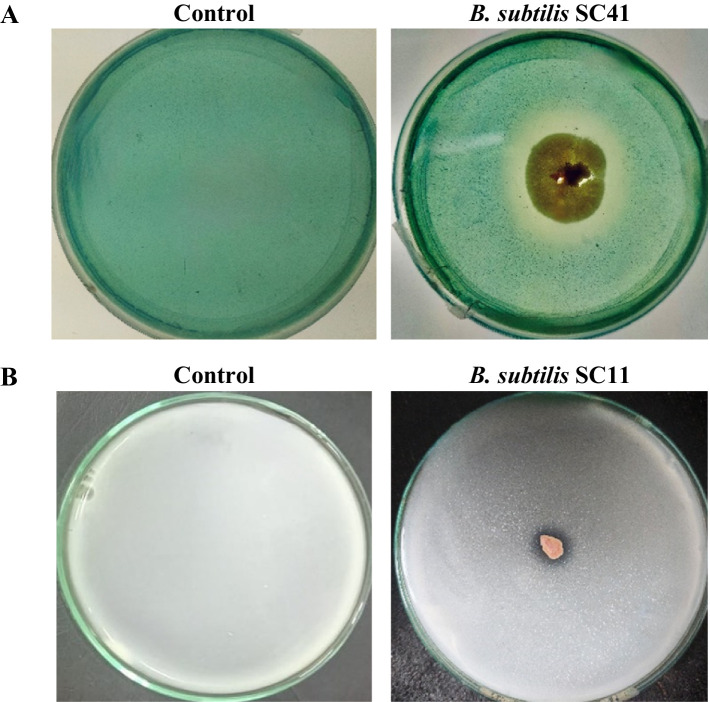
Representative examples. **(A)** Plate assay for the production of siderophore indicated by the change in medium color from blue to yellow/orange due to iron removal from CAS/HDTMA-Fe^+3^. **(B)** P-solubilization indicated by formation of a halo zone around the bacterial colony in Pikovskaya’s agar medium.

Out of the eight isolates, five isolates performed siderophore production in CAS agar media as indicated by a change of the medium color from blue to yellow-orange in the qualitative assay (Figure 3A). The quantitative assay indicated siderophore production ranging from 17.4 to 33.6% (Table 2). Only isolate *B. subtilis* SC11 was capable of P-solubilization in Pikovskaya’s agar medium (Table 1). P- solubilization was categorized based on the area of zone of clearance (Figure 3B). None of the eight bacterial isolates produced IAA.

### 3.5 Biocontrol of fusarium wilt on cotton plant using rhizospheric bacterial isolates

**Figure 4:**
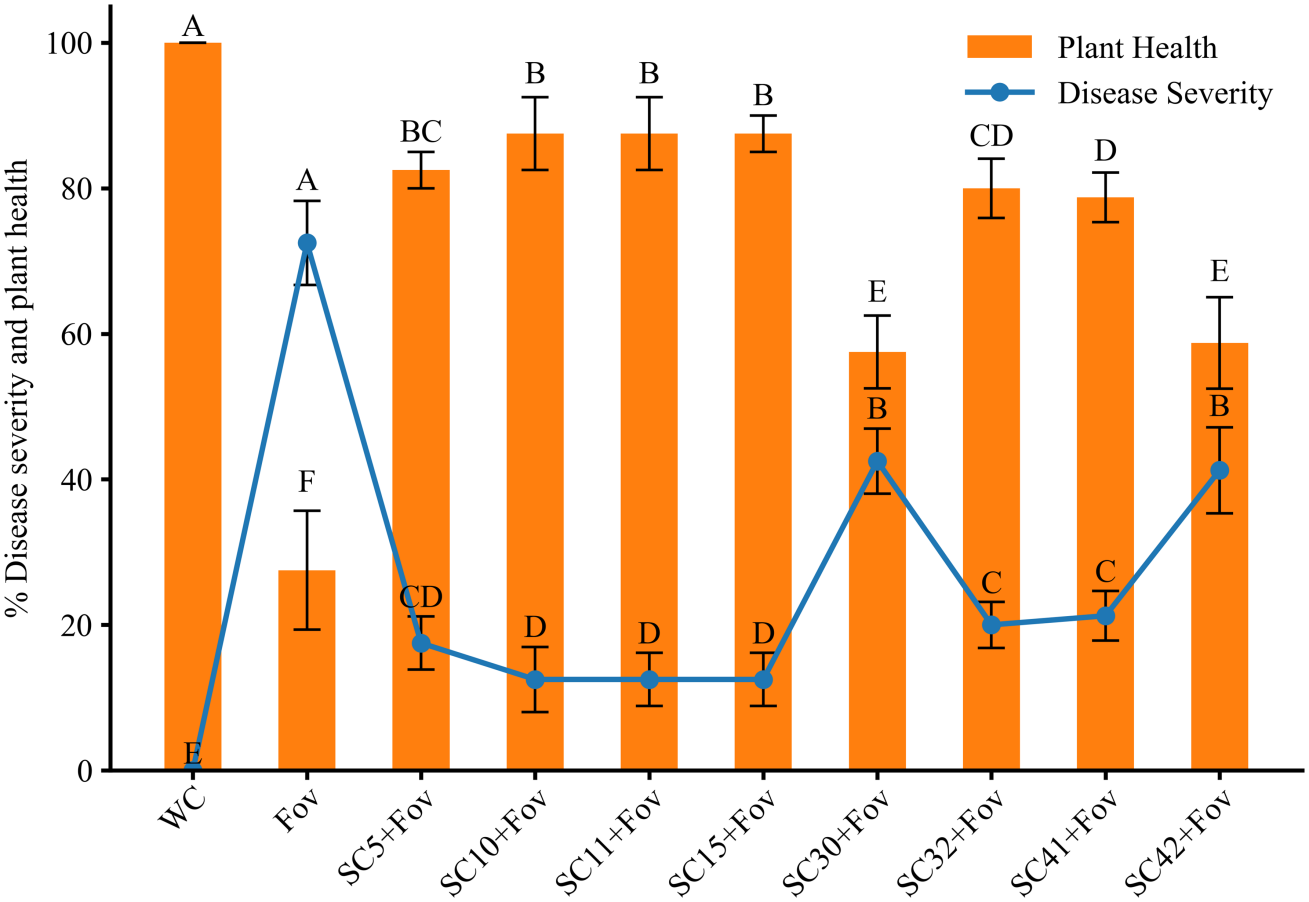
Effects of *Bacillus* isolates (SC5, SC10, SC11, SC15, SC30, SC32, SC41, and SC42) inoculation on disease severity and plant health in cotton under Fov stress. Values represent the average + standard deviation of two biological replicates (*n* = 16 in total). Significant differences between treatments are indicated by different letters. WC, water control; Fov, *Fusarium oxysporum* f.sp. *vasinfectum.*

**Figure 5:**
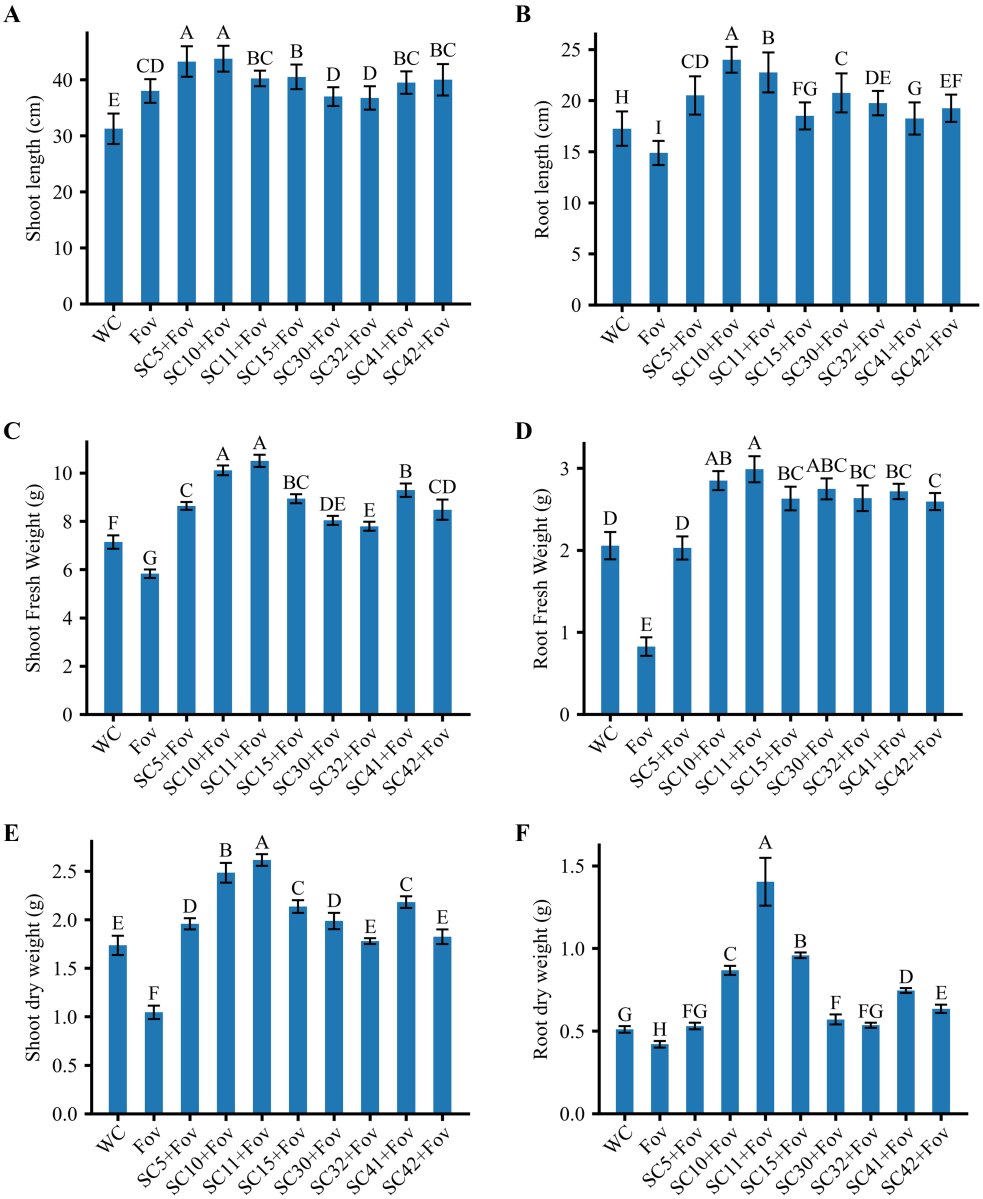
Effects of *Bacillus* isolates (SC5, SC10, SC11, SC15, SC30, SC32, SC41, and SC42) inoculation on cotton plants with Fov stress; **(A)** shoot length (*n* = 16), **(B)** root length (*n* = 16), **(C)** shoot fresh weight (*n* = 16), **(D)** root fresh weight (*n* = 16), **(E)** shoot dry weight (n = 8), and **(F)** root dry weight (*n* = 16). Values represent the average + standard deviation of two biological replicates (*n* = 8 or 16 in total). Significant differences between treatments are indicated by different letters. WC, water control; Fov, *Fusarium oxysporum* f.sp. *vasinfectum*.

In order to validate the biocontrol potential of the eight *Bacillus* isolates, greenhouse pot experiments were conducted to determine how well these bacteria reduce the severity of Fusarium wilt caused by Fov on cotton plants. Additionally, the isolates’ impact on cotton plant growth was investigated under Fov infection pressure. We found that all bacterial treatments reduced disease severity and additionally improved plant growth parameters significantly compared to plants treated with Fov alone. The disease severity percentage in treatments with *Bacillus* isolates *B. stercoris* SC5, *B. subtilis* SC10, SC11, SC41 and *B. halotolerans* SC15, SC32 presented themselves as considerably lower (12.5 ± 5 to 21.25 ± 2.5) compared to the control infected only with Fov (72.5 ± 5) (Figure 4). Moreover, *B. subtilis* SC10 and SC11, in particular, showed the overall highest enhancement in both shoot and root growth parameters (Figure 5). Compared to the Fov-inoculated control plants, application of *B. subtilis* SC10 and SC11 resulted in 15.8 and 5.3% longer shoots, 60 and 53.3% longer roots, 150 and 160% more shoot dry weight, 125 and 250% more root dry weight, respectively. Even compared to the uninoculated control, both isolates significantly increased shoot and root growth of Fov-inoculated plants. Although *B. stercoris* SC5, *B. halotolerans* SC15, and *B. subtilis* SC41 display a positive effect on plant health by combating Fov infestation, their performance does not surpass that of the most successful *B*. sublitis isolates SC10 and SC11. However, these strains’ ability to substantially decrease Fov severity while promoting better plant growth compared to the Fov-inoculated control suggests that they are also promising candidates for biocontrol applications against Fusarium wilt.

Conversely, treatments *B. halotolerans* SC30 and *B subtilis* SC42 showed marginal biocontrol effects with a disease severity percentage of 42.5 ± 5 and 41.25 ± 6, respectively. Although this equals a reduction of 41.38% (*B. halotolerans* SC30) and 43.10% (*B subtilis* SC42) compared to the Fov-inoculated control plants, it underlines the variability in *Bacillus* strains’ capacity to function as biocontrol agents.

### 3.6 Effect on oxidative stress and antioxidant activities

**Figure 6:**
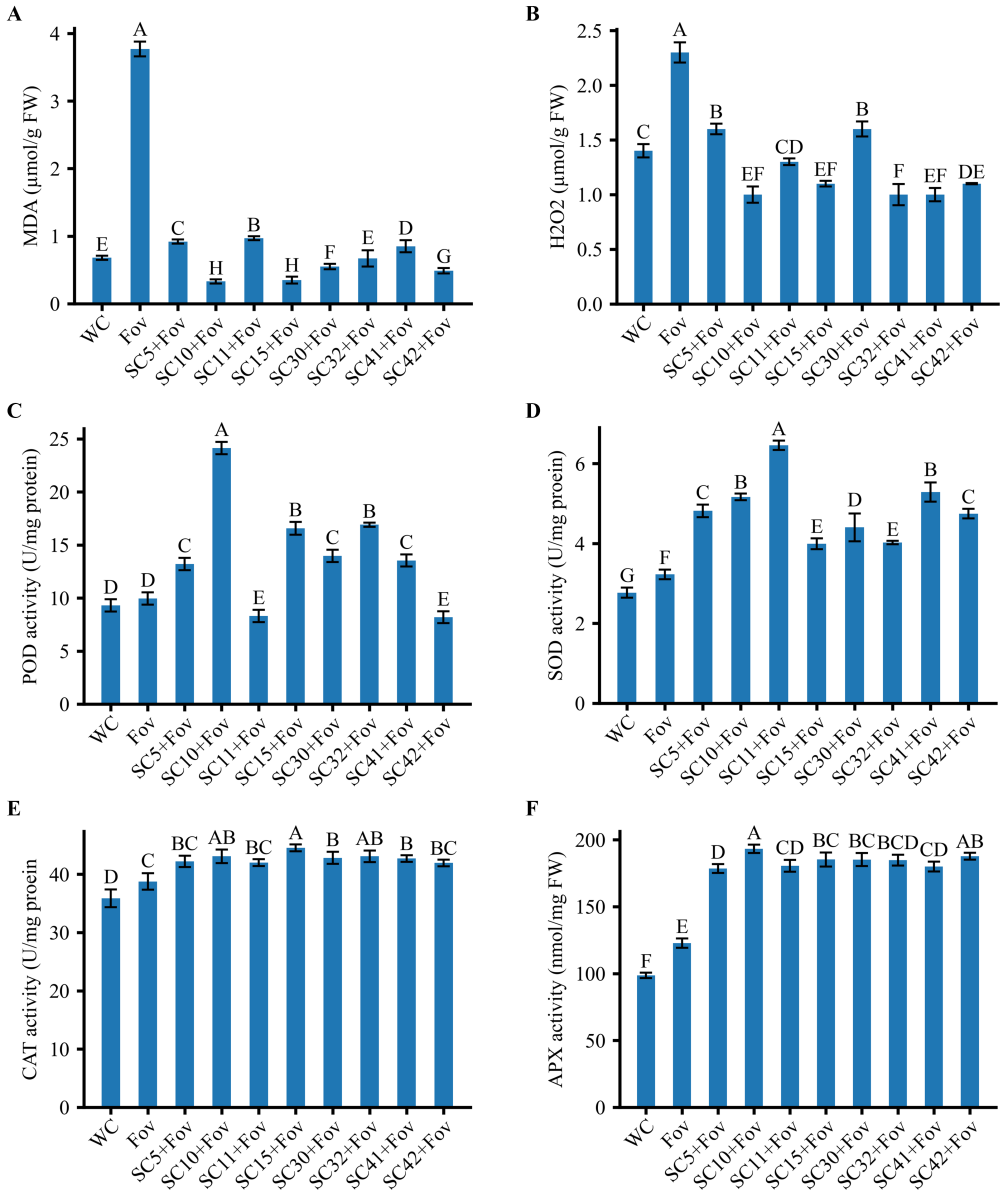
Effect of *Bacillus* isolates (SC5, SC10, SC11, SC15, SC30, SC32, SC41, and SC42) on ROS and antioxidant activities of cotton **(A)** malondialdehyde (MDA), **(B)** H_2_O_2_, **(C)** Peroxidase (POD) activity, **(D)** Superoxide dismutase (SOD) activity, **(E)** Catalase (CAT) activity, **(F)** Ascorbate peroxidase (APX) activity. Values represent the average + standard deviation of two biological replicates (*n* = 24 in total). Significant differences between the treatments are indicated by different letters. WC, water control; Fov, *Fusarium oxysporum* f.sp. *vasinfectum*.

Next, oxidative stress markers were investigated to obtain insights into the physiological impact of Fov infection in plants, particularly when treated with *Bacillus* strains. The analysis of oxidative stress markers in plants subjected to Fov infection revealed a significant elevation in the levels of H_2_O_2_ and MDA, which are indicative of cellular damage due to oxidative stress. Specifically, the Fov infected plant exhibited higher concentrations of MDA and H_2_O_2_ with an approximate increase of 455 and 69%, respectively as compared to the control plants treated with water only (Figure 6). Plants within the WC group maintained baseline levels of H_2_O_2_ and MDA, reflecting the absence of (pathogenic) stress and indicating normal physiological conditions. In contrast, the increase of H_2_O_2_ and MDA in the Fov group suggests that the fungal infection prompted an oxidative burst, a typical plant response that typically stimulates antioxidant enzyme activities as a part of the plant’s defensive response to plant pathogens and oxidative stress.

This is validated by the observed increase in POD, SOD, CAT, and APX activities by 6.9, 16.6, 8.1, and 24.4%, respectively in the infected control compared to the water-treated control.

Application of the eight *Bacillus* strains to Fov-inoculated cotton plants significantly reduced lipid peroxidation (74.4 to 91.4% reduction compared to the Fov control) and H_2_O_2_ production (29.4 to 58.4% reduction compared to the Fov control) while causing a considerable increase in POD in some treatments including *B. stercoris* SC5, *B. subtilis* SC10 (with highest increase of 142.61% compared to Fov control), *B. halotilerans* SC15, SC30, SC32, *B. subtilis* SC42 and reduction in *B. subtilis* SC11 (16.58%) and SC42 (17.69%) compared to Fov control. SOD was increased in all *Bacillus* treatments (66.63% in SC32 + Fov to 100% in SC11 + Fov compared to Fov control). A moderate increase in CAT (8.39% in SC11 + Fov to 23.53% in SC15 + Fov compared to Fov control) and APX (10.17% in SC41 + Fov to 59.75% in SC10 + Fov) antioxidant activities was observed compared to the Fov-inoculated control plants.

**Figure 7:**
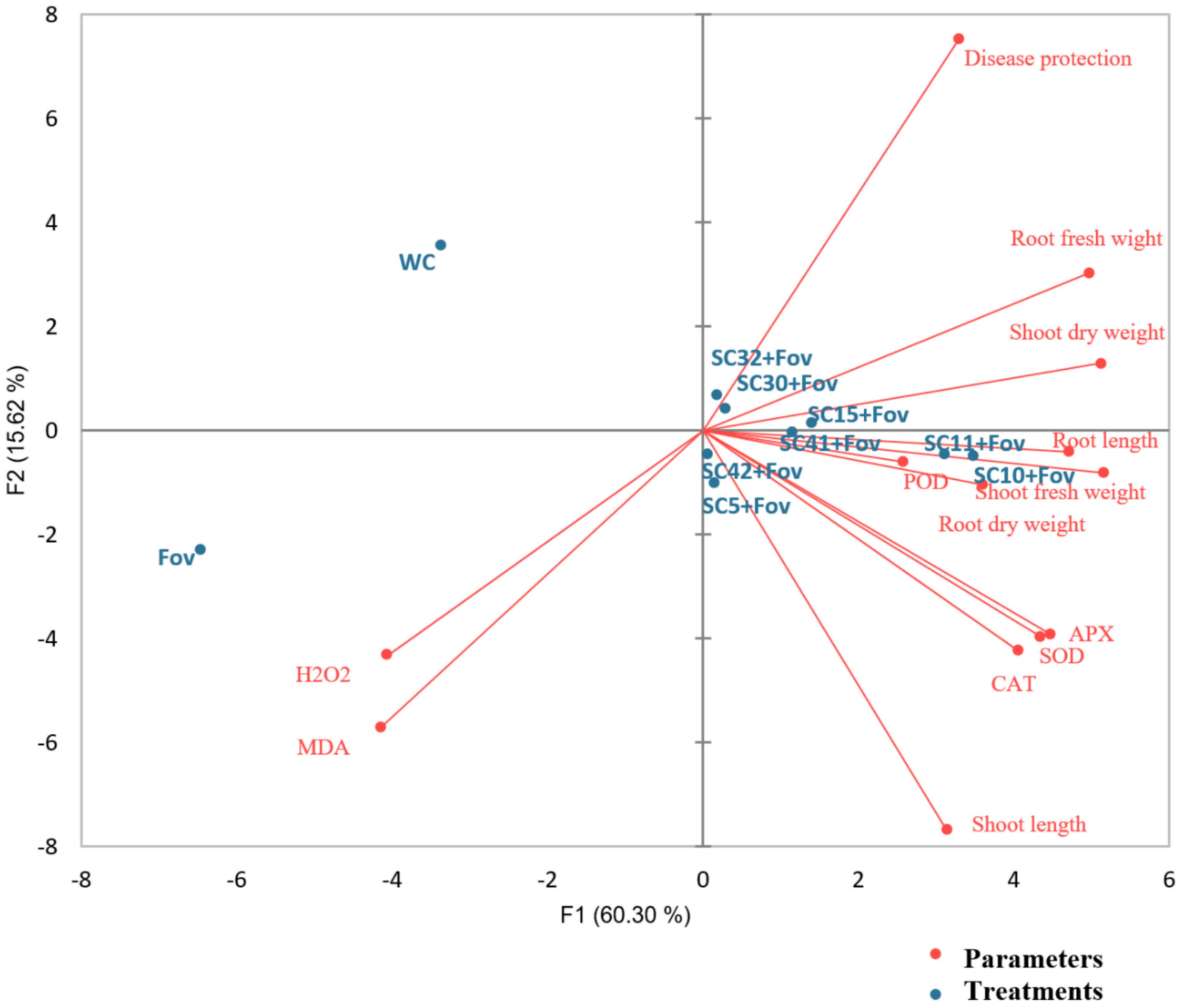
Principal component analysis biplot of the effects of the *Bacillus* isolates (SC5, SC10, SC11, SC15, SC30, SC32, SC41, and SC42) on reactive oxygen species (ROS), antioxidants, and growth parameters of cotton plants when applied during *Fusarium oxysporum* f.sp. *vasinfectum* (Fov) stress. WC, water control; H_2_O_2_, hydrogen peroxide; MDA, malondialdehyde; POD, peroxidase; APX, ascorbate peroxidase; SOD, superoxide dismutase; CAT, catalase.

These elevated antioxidant levels suggest that all the *Bacillus* treatments greatly enhanced the defensive response of the plants against oxidative stress caused by the fungal pathogen. Data of all agronomical parameters, ROS, and antioxidants are also represented in Figure 7 using principle component analysis biplot to explore relationships between different treatments and parameters. The treatments appear to cluster into distinct groups. The no-treatment WC is separated from the Fov-infected control and most of the *Bacillus* treatments. The PCA biplot illustrates the relationships between different treatments and physiological parameters in cotton plants under Fov infection. The first two principal components (F1 = 60.30%, F2 = 15.62%) together explain 75.92% of the total variance. The *Fov*-infected plants show a strong association with oxidative stress markers (H₂O₂ and MDA), indicating enhanced lipid peroxidation and ROS accumulation. In contrast, disease protection, root fresh weight, shoot dry weight, and root dry weight are positively correlated, suggesting that these traits contribute to plant resistance. Antioxidant enzymes (APX, SOD, and CAT) cluster together, highlighting their role in mitigating oxidative stress. Treatments such as SC10 + Fov and SC11 + Fov are closely associated with root and shoot growth parameters, indicating a potential resistance effect, while WC remains distant from stress markers, signifying a healthier state. The analysis reveals that *Fov* infection negatively affects plant growth through oxidative damage, while specific treatments promote tolerance by enhancing antioxidant defense and maintaining biomass production. This suggests that the *Bacillus* treatments have a measurable effect on the plant’s response to Fov infection.

## 4 Discussion

The results of this study highlight the significant biocontrol potential of *Bacillus* species against Fusarium wilt, a debilitating disease that severely impacts the productivity of a wide range of economically important crops. This study adds significantly to the growing body of data supporting the use of biological control agents as a sustainable and environmentally friendly alternative to conventional chemical fungicides. In this study, we evaluated the biocontrol potential of rhizobacteria isolated from the rhizosphere of cotton plants infected with fungal pathogens. We succeeded in discovering *Bacillus* isolates with antagonistic activity against Fov and additionally against *F. graminearum*, *L. maculans* and *C. becticola in vitro* and biocontrol activity against Fov in greenhouse pot experiments with cotton plants. Eight isolates showed the highest antifungal activity, reducing fungal growth by up to 93% *in vitro* (Table 1). Molecular characterization based on 16S rRNA revealed that all eight isolates belonged to the genus *Bacillus*. Members of the genus *Bacillus* stand out as the main group of rhizospheric bacteria utilized for the biological control of plant-pathogenic fungi (Boulahouat et al., 2023). The effectiveness of these bacteria in biocontrol is due to their production of diverse secondary metabolites and their capacity for swift propagation, both of which allow for their effective incorporation into biocontrol approaches (Kiesewalter et al., 2021). The prevalence of *Bacillus* isolates with biocontrol potential can be attributed to several factors:, their ubiquity and adaptability, their established role as biocontrol agents producing various antimicrobial compounds, LB’s non-selectivity favoring fast-growing bacteria, Bacillus’ robust spore-forming ability and other potential sampling bias (Fira et al., 2018). The use of different isolation methods and media could reveal additional biocontrol agents.

The eight isolates were further evaluated for biocontrol and plant growth promoting activities such as hydrolytic enzyme production, siderophore, P solubilization, and IAA. In hydrolytic enzymes, chitinase can break down chitin, a major structural component of fungal cell walls (Veliz et al., 2017). By degrading pectin, pectinase enzymes can help the bacteria colonize plant tissues and potentially compete with fungi for space and nutrients (Riseh et al., 2024). While cellulose is not a major component of most fungal cell walls, some fungi produce cellulose in certain structures. Cellulase can degrade these structures, potentially affecting fungal growth and development (Brown Jr, 2004). PGPR cellulases primarily target dead organic matter, which increases nutrient availability for plants, and generally do not degrade intact plant cell walls. This is because plant cellulose is often protected by lignin and other structural components that PGPR cellulases cannot easily break down (McKee and Inman, 2019). Protease enzymes can target fungal cell wall proteins, contributing to cell wall weakening and disruption (Dimkić et al., 2022). Urease activity in bacteria plays several crucial roles, primarily related to nitrogen metabolism, pathogenicity, and environmental interactions (Pei et al., 2024). Siderophores are iron-chelating compounds produced by bacteria that contribute to the biocontrol of fungal pathogens by sequestering iron making it unavailable to fungi, but improving iron availability for plants and thus promoting plant growth. Furthermore, in some cases, they have direct antifungal activity, (by) and act synergistically with other biocontrol mechanisms (Elshahat et al., 2016). Improved P nutrition can strengthen plant defense mechanisms, making them more resistant to fungal pathogens (Huber and Haneklaus, 2007). A healthy, vigorously growing plant is generally better equipped to withstand pathogen attacks. The production of IAA, a key auxin hormone, is commonly associated with the ability of plant growth-promoting rhizobacteria to enhance plant root growth and overall vigor. Bacteria with antagonistic activity are known to play a crucial role in regulating plant growth and development through different mechanisms (Meena et al., 2020). In our study, no rhizobacteria was producing IAA although these rhizobacteria are good antagonists of fungi.

Although *in vitro* assays are valid in screening for antifungal capabilities, it is crucial to evaluate the biocontrol efficacy of PGPR through *in vivo* studies. Therefore, for validation, a greenhouse experiment was conducted using all *Bacillus* isolates to counteract Fov infection of cotton plants. The results showed that Fov infection decreased root and shoot fresh and dry weights compared to the uninfected control. All isolates suppressed Fusarium wilt from 41.30 to 82.7%. In particular, *B. subtilis* isolates SC10 and SC11 showed outstanding performance in increasing biomass and root development of cotton plants. Our findings on *B. subtilis* SC11, which possesses high siderophore production, strong urease activity, and P solubilization capabilities, indicate its potential as a highly effective biocontrol agent for plant growth promotion. The combined effects of siderophore production, urease activity, and P solubilization can act synergistically to promote plant growth and suppress fungal pathogens (Sritongon et al., 2023). For example, iron and phosphorus deficiency can weaken plants, making them more susceptible to disease. By increasing the availability of these nutrients, SC11 can improve plant health and make plants more resistant to fungal attack. Our results also indicate that Fov-mediated oxidative stress affects the activity of antioxidant enzymes in cotton plants. The observed increase in MDA levels indicated oxidative stress-induced membrane damage caused by Fusarium wilt in cotton plants. This increase in MDA, a marker of oxidative stress and cell membrane damage, is consistent with previous research. Notably, cotton plants inoculated with antagonistic *Bacillus* strains exhibited significantly reduced MDA levels compared to those inoculated with Fov alone, suggesting that these rhizobacterial strains mitigate the deleterious effects of Fov. The reduction in MDA levels due to inoculation resulted in reduced oxidative damage, thereby promoting plant growth.

In this study, Fov appeared to activate antioxidant enzymes such as POD, SOD, CAT, and APX in exposed cotton plants, revealing a protective response to Fov-mediated oxidative stress. This is consistent with previous research, in which was found that different plants increased their antioxidant enzyme activities to scavenge ROS generated by fungal stress (Zhang et al., 2004). After the application of PGPR, there was a further increase in the activities of these enzymes in Fov-infected plants, indicating the beneficial role of plant-microbe interactions under fungal stress. These interactions help plants to cope with the harmful effects of Fov by enhancing their antioxidant defense mechanisms.

Antioxidant enzymes, including SOD, POD, CAT, and APX play a critical role in protecting plants from oxidative damage caused by ROS. SOD catalyzes the conversion of superoxide radicals to H₂O₂ and oxygen, thereby reducing the damaging effects of ROS. CAT then rapidly breaks down H₂O₂ into water and oxygen, preventing its accumulation. Similarly, APX uses ascorbate as an electron donor to reduce H₂O₂ to water, thereby maintaining cellular redox balance. POD also contributes to using various electron donors to scavenge H₂O₂. Together, these enzymes form an integrated defense system to mitigate oxidative stress, protect cellular components, and ensure metabolic homeostasis (Hasanuzzaman et al., 2020). In our study cotton plants inoculated with Fov and treated with *Bacillus* isolates showed higher SOD, POD (except SC11 and SC42), CAT, and APX activity compared to those without treatment (WC) and those inoculated with Fov alone. Our results are in line with studies in so far that an increase in various antioxidants is a significant plant-resistance feature against plant pathogens as elevated levels of antioxidant enzyme activity protected cotton plants from the oxidative damage induced by fungal infection and mitigated the adverse effects of lipid peroxidation caused by ROS (De Gara et al., 2003). Therefore, it can be concluded that the described *Bacillus* isolates combat Fov-induced stress and have the ability to induce plant defense mechanisms.

Future field trials will validate whether the isolates identified in this study, particularly *B. subtilis* SC10 and SC11, effectively control Fov and promote plant growth in practical agricultural conditions. An interesting aspect to investigate as well is whether an individual isolate is sufficiently effective or whether a consortium of different bacteria is preferable. Recently, a consortium of three *Bacillus* isolates was shown to promote plant grown of *Vicia faba* better and to control *Fusarium oxysporum* more efficiently than the individual strains (El-Sersawy et al., 2021). Moreover, a comparison between the performance of the bacteria and a commercially available synthetic chemical seed treatment is an interesting aspect to look into and necessary to gain acceptance.

## 5 Conclusion

The experimental data reveal that all eight bacterial isolates SC5, SC10, SC11, SC11, SC30, SC32, SC41, and SC42 that were originally isolated from the cotton rhizosphere of Fov and subsequently selected for their fungal activity against Fov also show beneficial traits associated with plant growth promotion and plant protection against other fungi. Particularly, *B. subtilis* SC10 and *B. subtilis* SC11, when applied to cotton plants against Fov, significantly reduced Fov infection, enhanced plant growth, and strengthened the defense system against Fusarium wilt. The *B. subtilis* SC10 treatment stands out with the highest shoot growth and biomass enhancement, suggesting an overall improvement in aboveground plant vitality. *B. subtilis* SC11 showed exceptional performance in promoting root growth and had the highest activity of CAT, APX, and SOD key antioxidant enzymes, which are critical for mitigating oxidative stress caused by pathogen attacks. The lower disease severity observed for the treatments with the *Bacillus* isolates, particularly *B. subtilis* SC10 and SC11, further supports their potential as effective biocontrol agents. The lower MDA and higher antioxidant levels in these treatments provide additional support for their protective effects. These isolates are good candidates for the generation of versatile bio-formulations with the capacity to enhance plant growth and ensure plant health. These formulations could then be commercially leveraged for integrated plant nutrient and pathogen management, thereby endorsing practices that facilitate sustainable agriculture.

## Data Availability

The datasets presented in this study can be found in online repositories. The names of the repository/repositories and accession number(s) can be found below: GenBank accession numbers PV147758 to PV147765. https://www.ncbi.nlm.nih.gov/nuccore/?term=PV147758:PV147765[accn].
