# Supplementary material for: Rhizospheric Bacillus isolates control Fusarium wilt on cotton and enhance plant biomass and root development
Source: Front Microbiol. 2025 May 2;16:1580937. doi: 10.3389/fmicb.2025.1580937 (PMC12081333; doi:10.3389/fmicb.2025.1580937)
Supplement: Supplementary file 1 [file Data_Sheet_1.zip › Figure 2.docx]

Figure 2

| **A** |  | **Control** |  | **B** | **Control** |  |  |
| --- | --- | --- | --- | --- | --- | --- | --- |
|  |  | **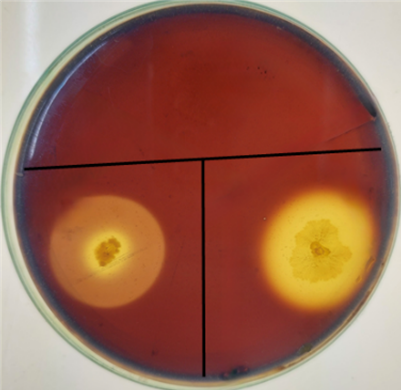**  ***B. halotolerans* SC15** |  | ***B. stercoris* SC5** | **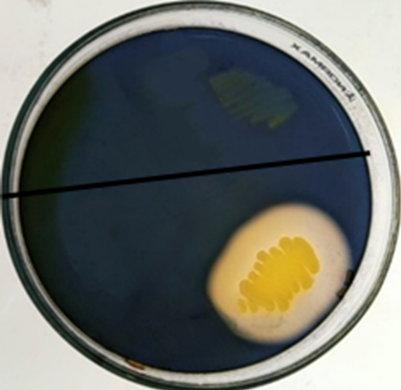** | **E** | **Control**  ***B. stercoris* SC5**  **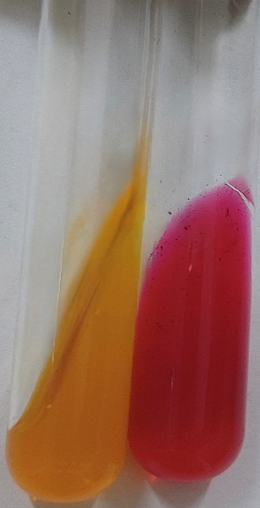** |
|  |  |  |  |  | ***B. subtilis* SC11** |  |  |
| **C** |  | **Control** |  | **D** |  |  |  |
|  |  | **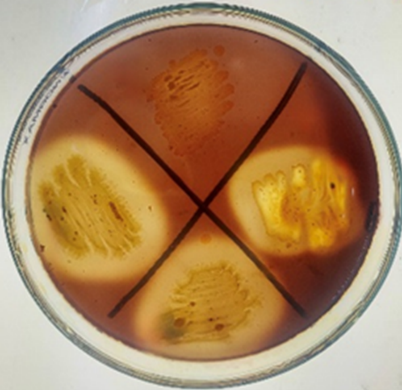**  ***B. subtilis* SC11** |  | ***B. stercoris* SC5** | **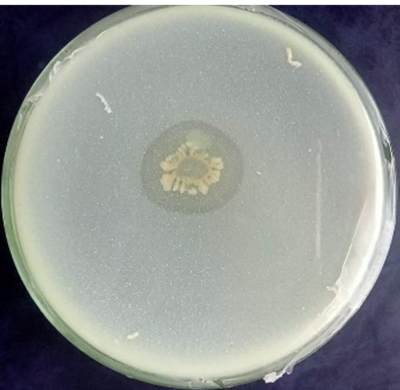** |  |  |
|  |  | ***B. subtilis* SC41** |  |  | ***B. halotolerans* SC15** |  |  |
