# Supplementary figures and images for: Rhizospheric Bacillus isolates control Fusarium wilt on cotton and enhance plant biomass and root development
Source: Front Microbiol. 2025 May 2;16:1580937. doi: 10.3389/fmicb.2025.1580937 (PMC12081333; doi:10.3389/fmicb.2025.1580937)

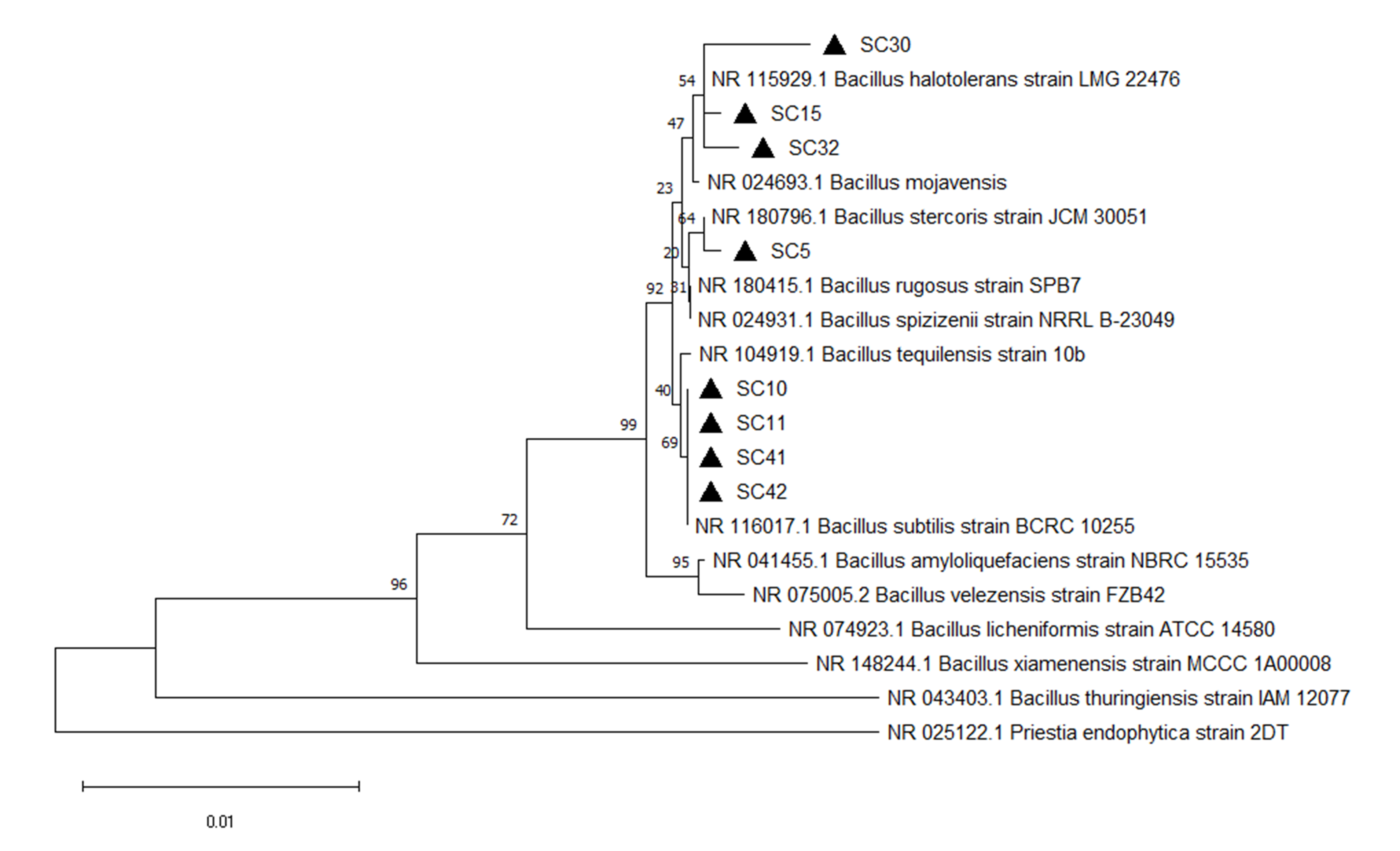

Supplement: Supplementary file 1 [file Data_Sheet_1.zip › Figure 1.tif]

Figure 3

| **A** | **Control** | ***B. subtilis* SC41** |
| --- | --- | --- |
|  | 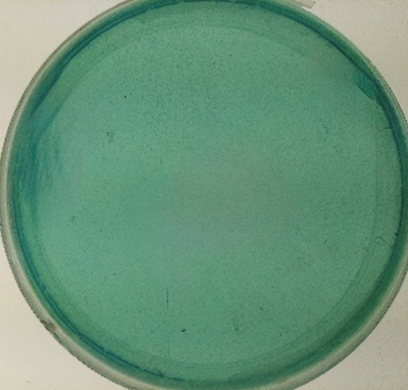 | 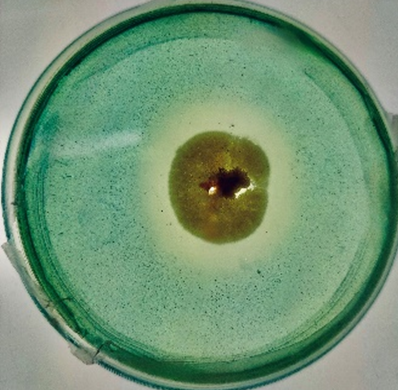 |
| **B** | **Control** | ***B. subtilis* SC11** |
|  | 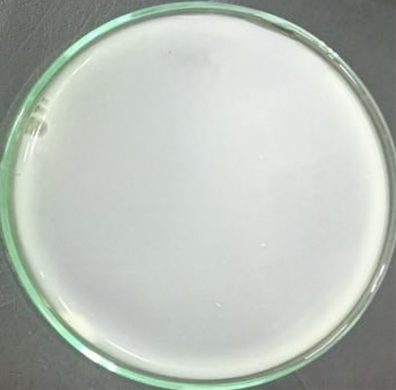 | 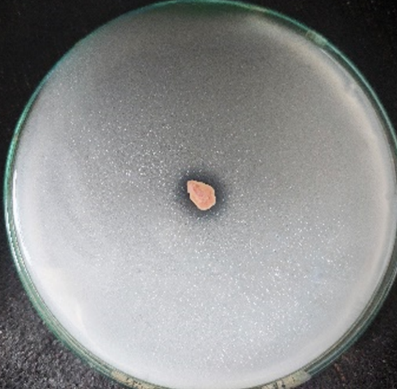 |

Supplement: Supplementary file 1 [file Data_Sheet_1.zip › Figure 3.docx]

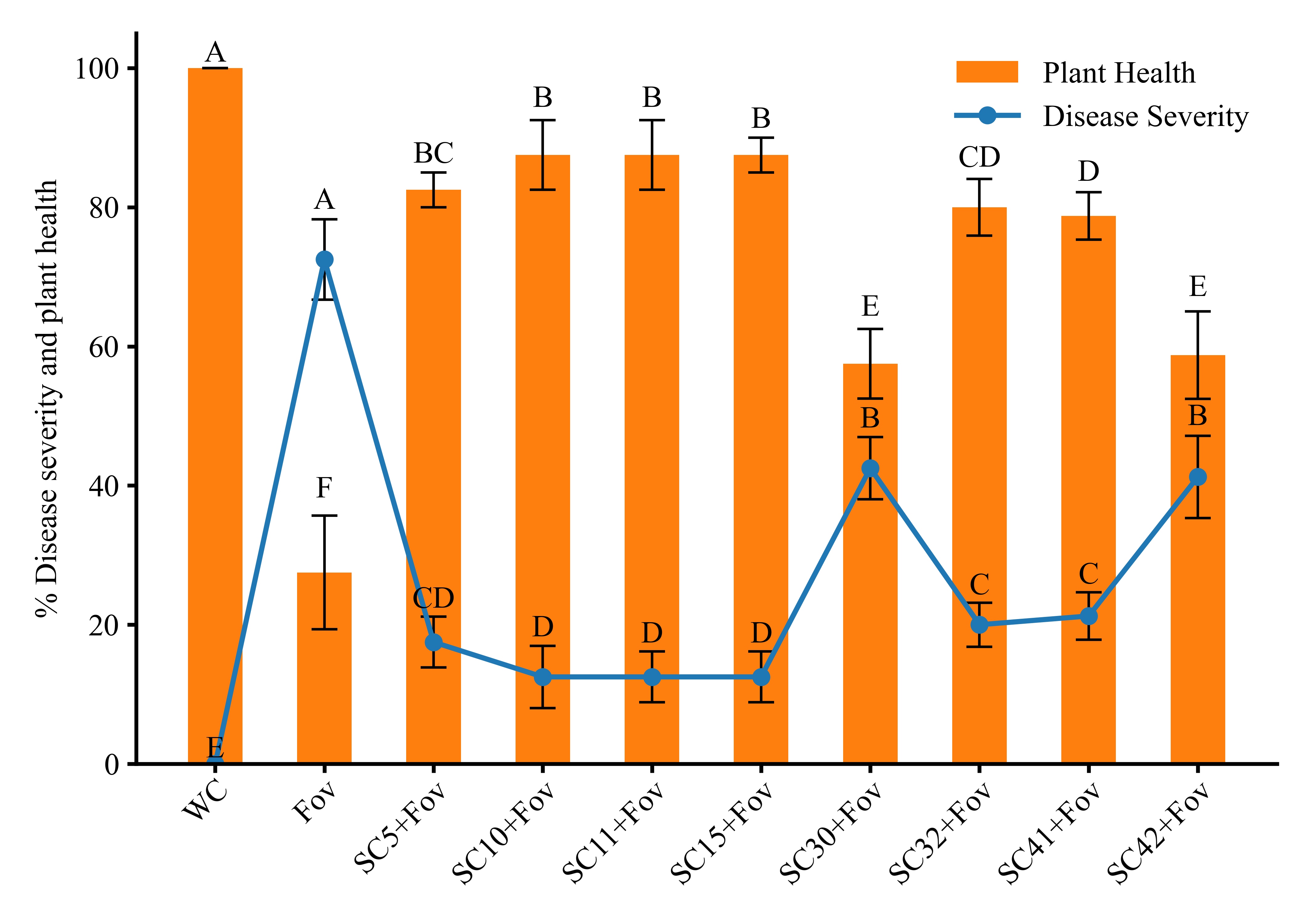

Supplement: Supplementary file 1 [file Data_Sheet_1.zip › Figure 4.tiff]

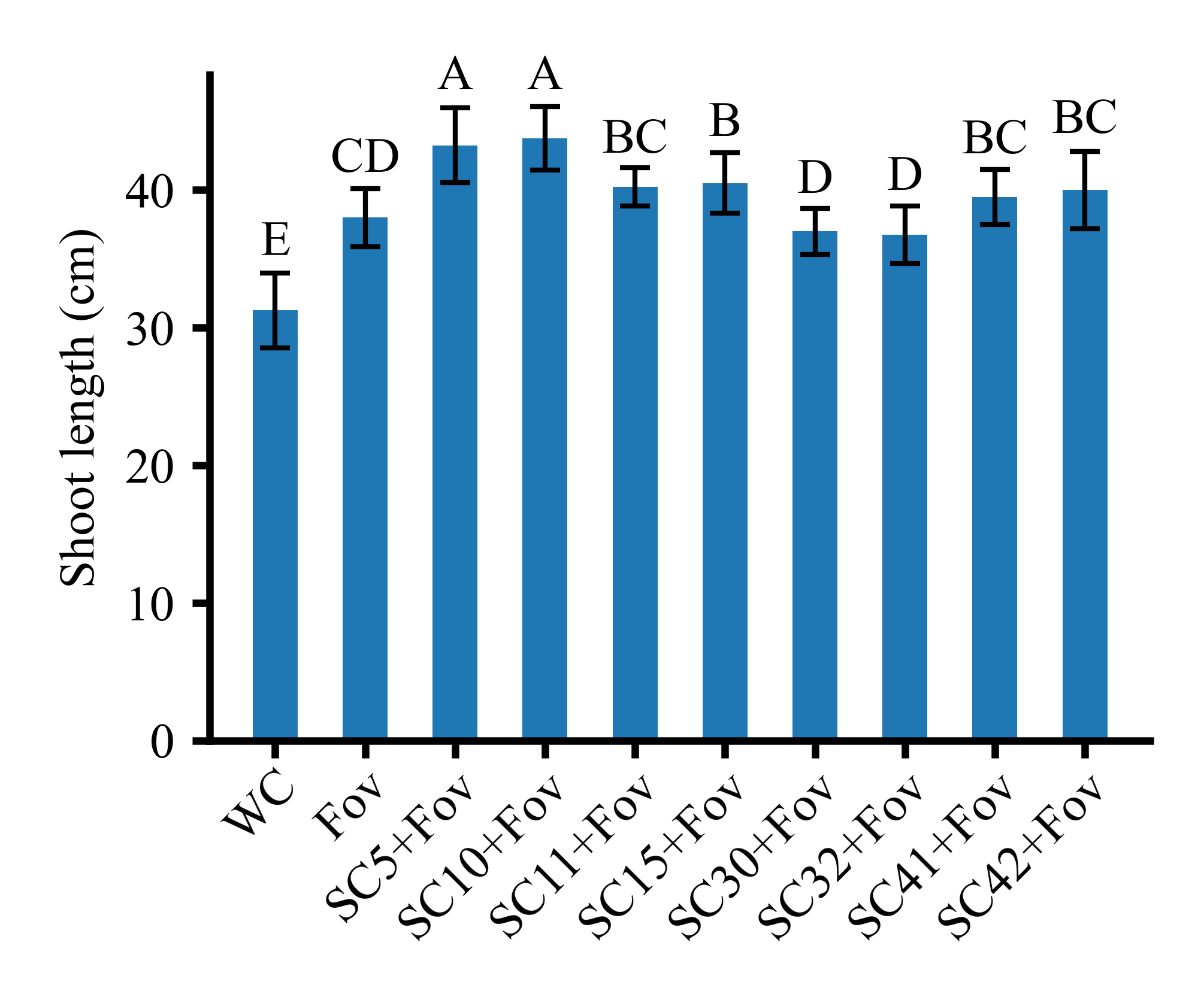

Supplement: Supplementary file 1 [file Data_Sheet_1.zip › Figure 5A.tiff]

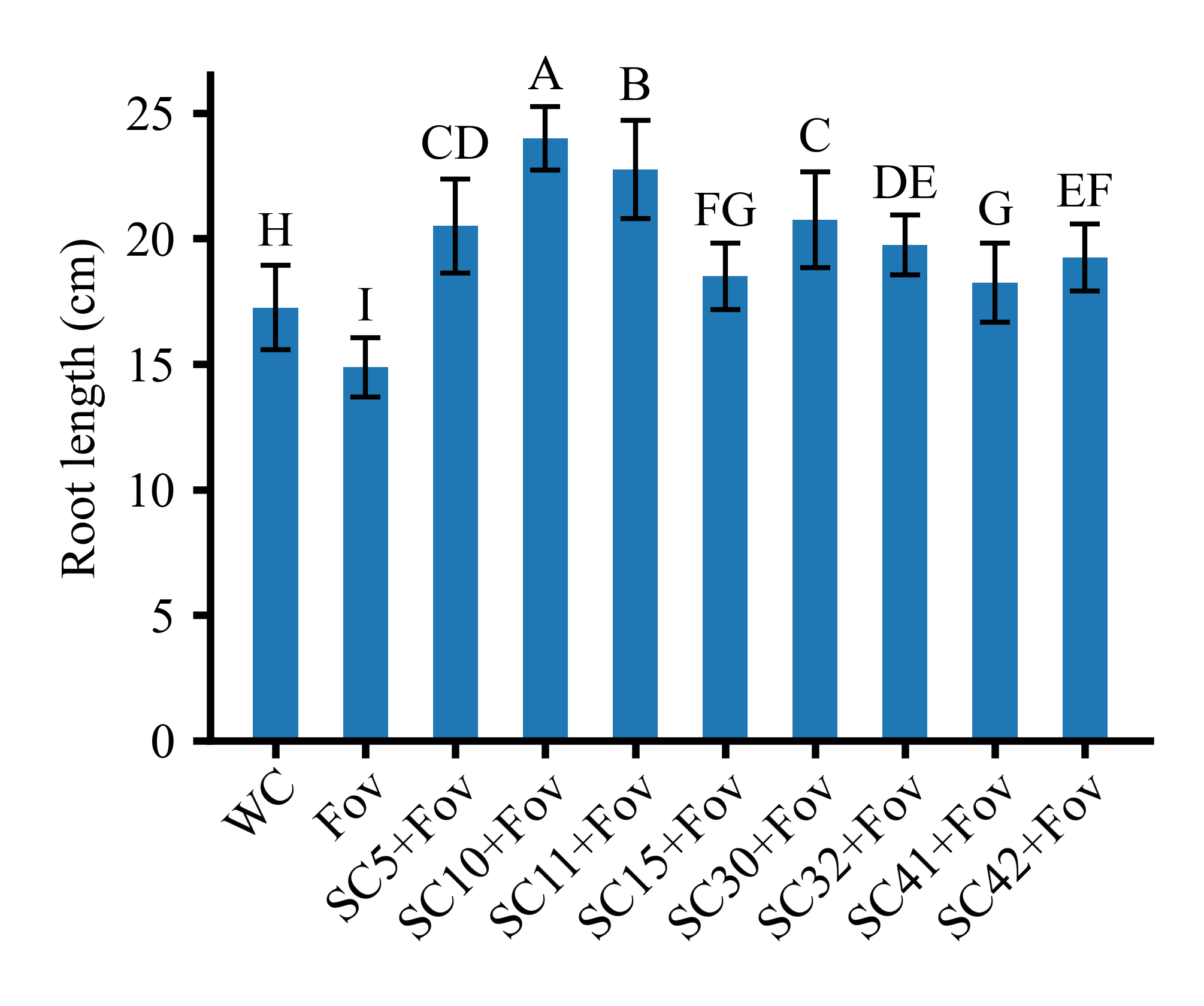

Supplement: Supplementary file 1 [file Data_Sheet_1.zip › Figure 5B.tiff]

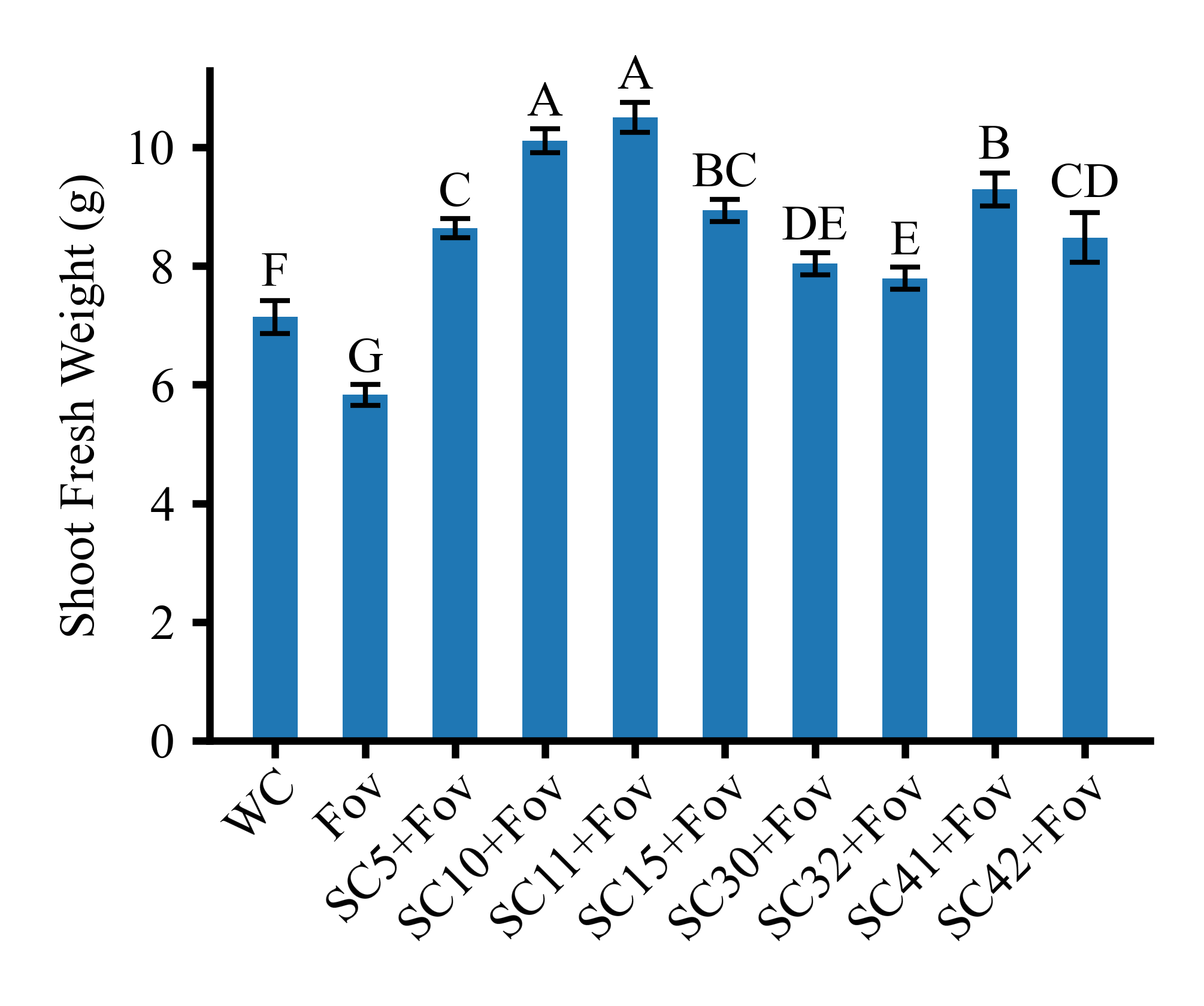

Supplement: Supplementary file 1 [file Data_Sheet_1.zip › Figure 5C.tiff]

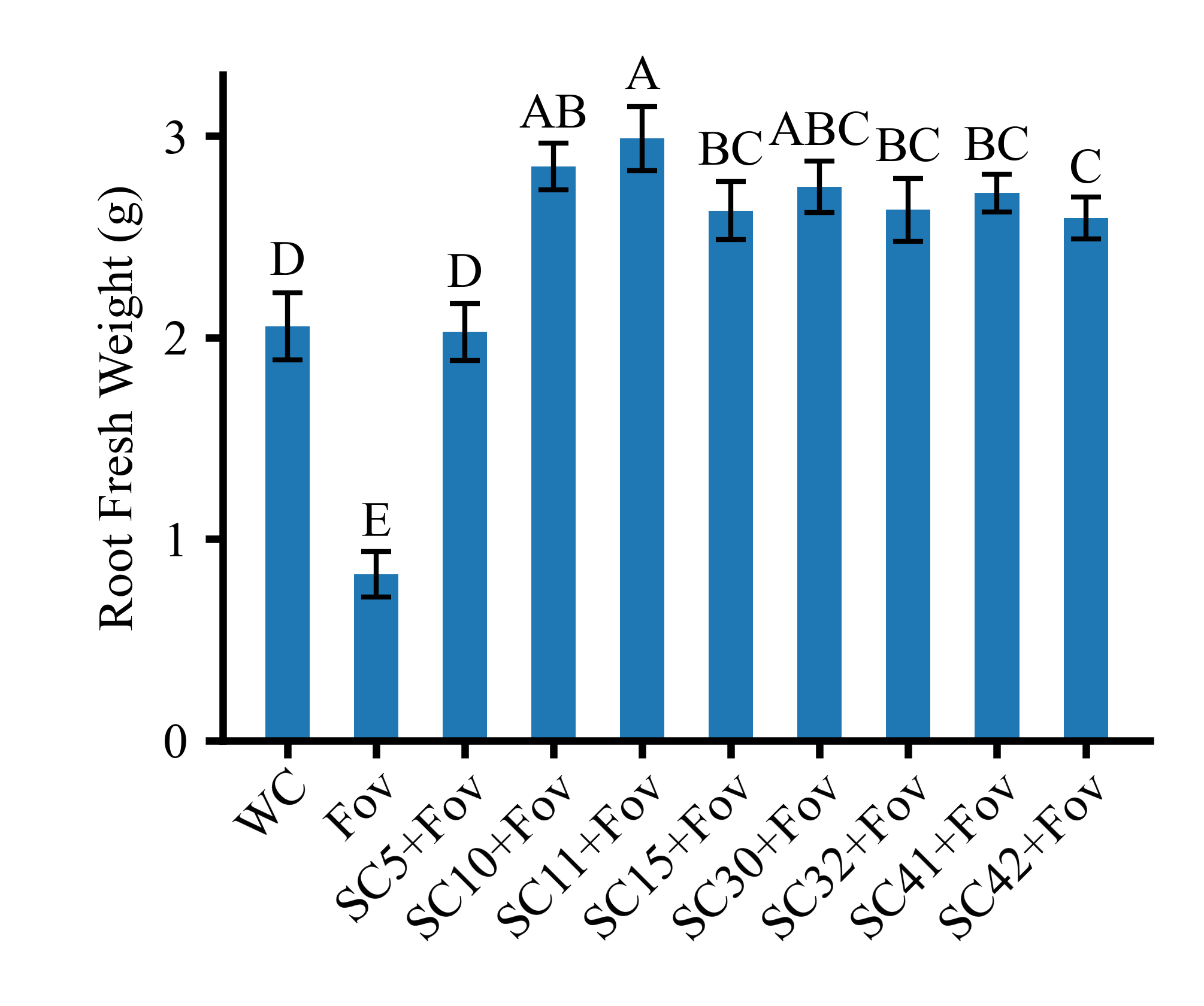

Supplement: Supplementary file 1 [file Data_Sheet_1.zip › Figure 5D.tiff]

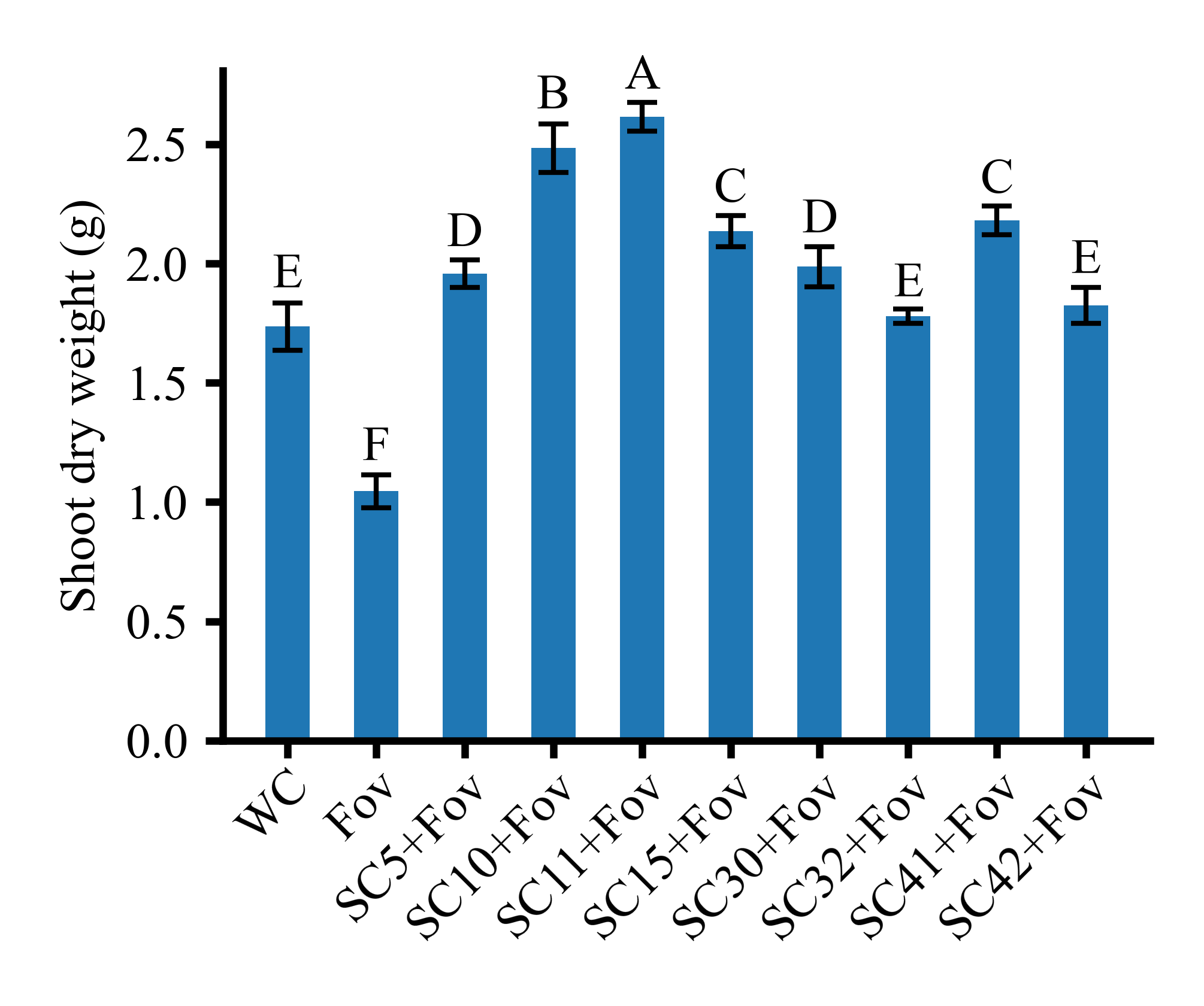

Supplement: Supplementary file 1 [file Data_Sheet_1.zip › Figure 5E.tiff]

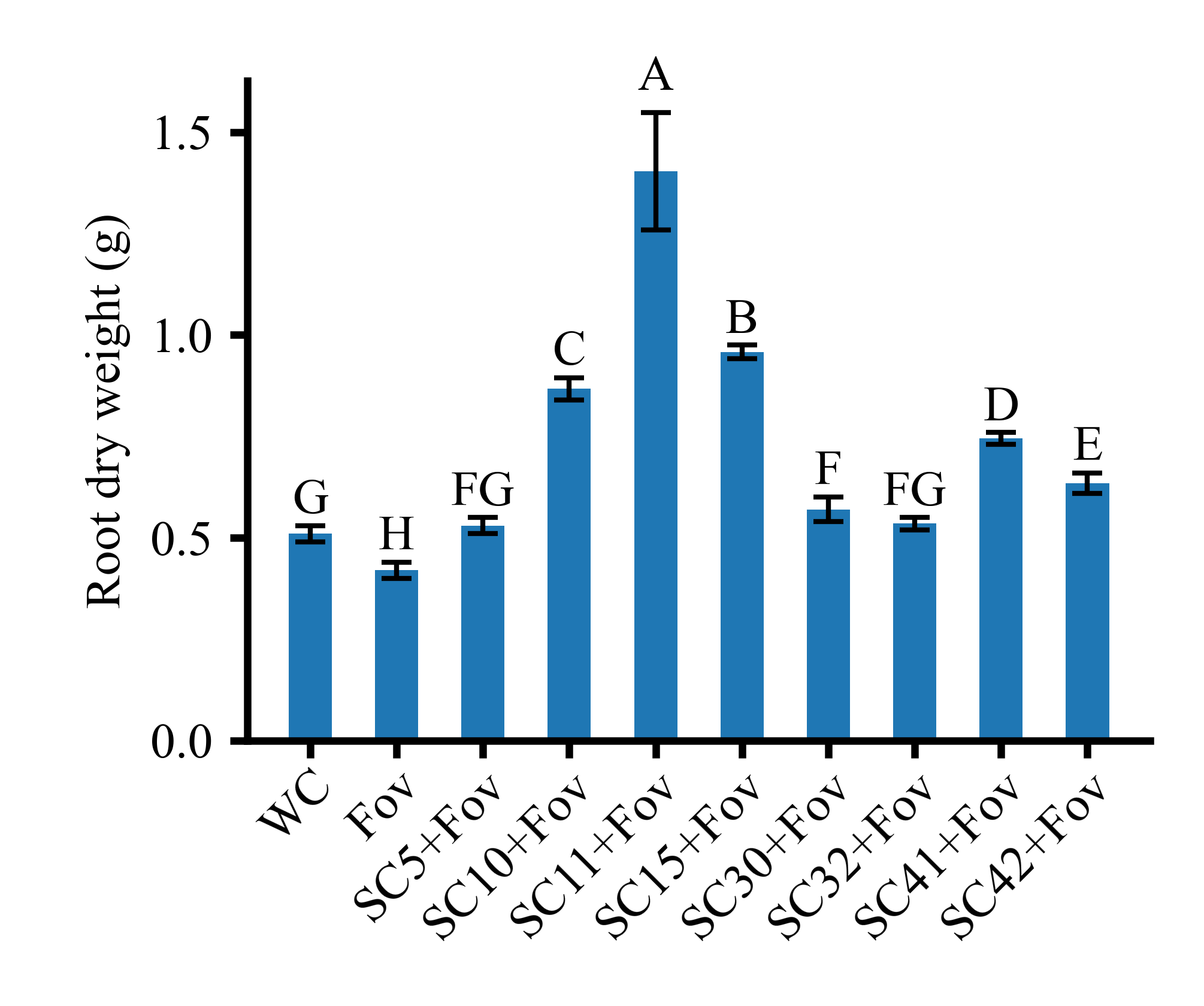

Supplement: Supplementary file 1 [file Data_Sheet_1.zip › Figure 5F.tiff]

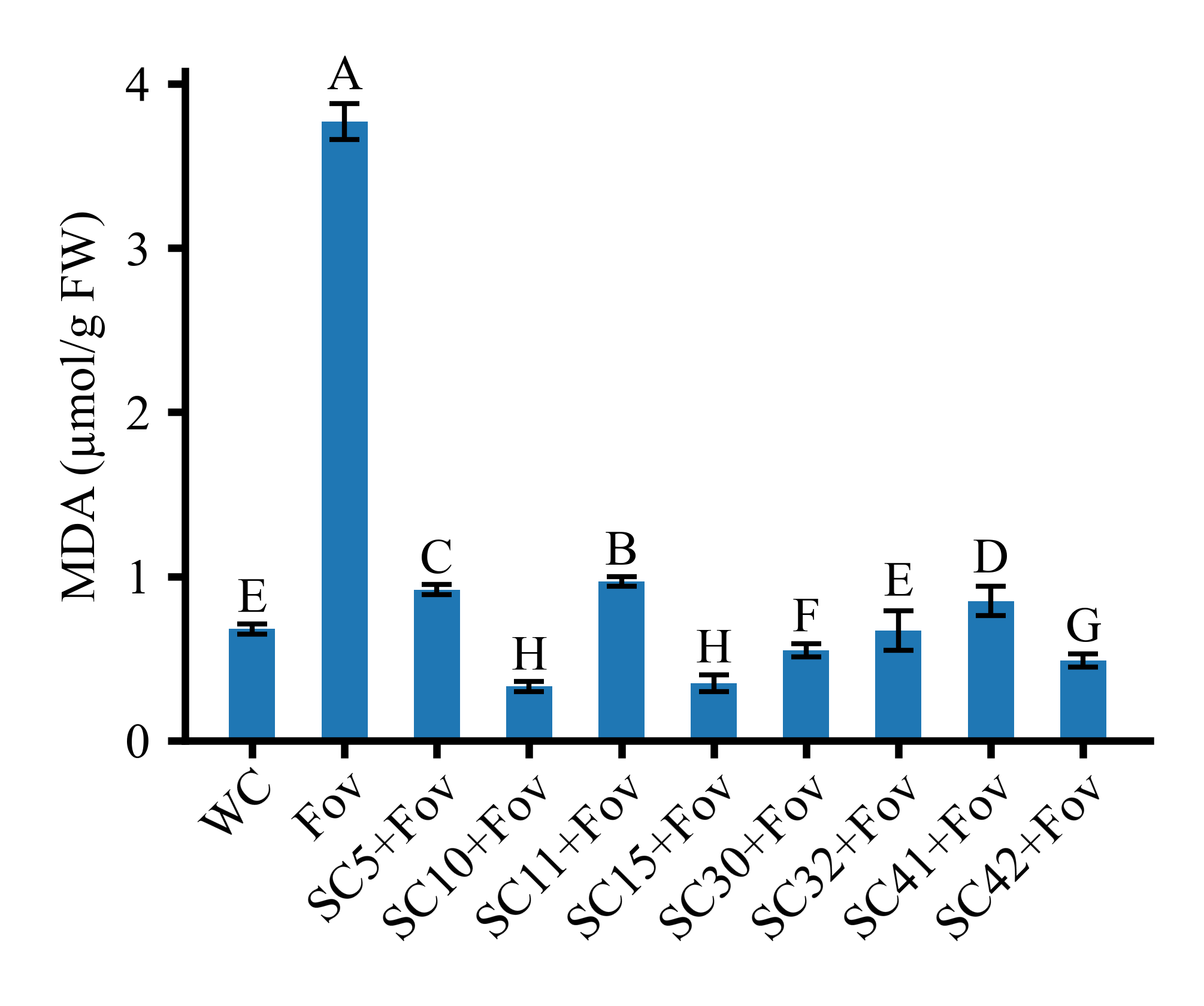

Supplement: Supplementary file 1 [file Data_Sheet_1.zip › Figure 6A.tiff]

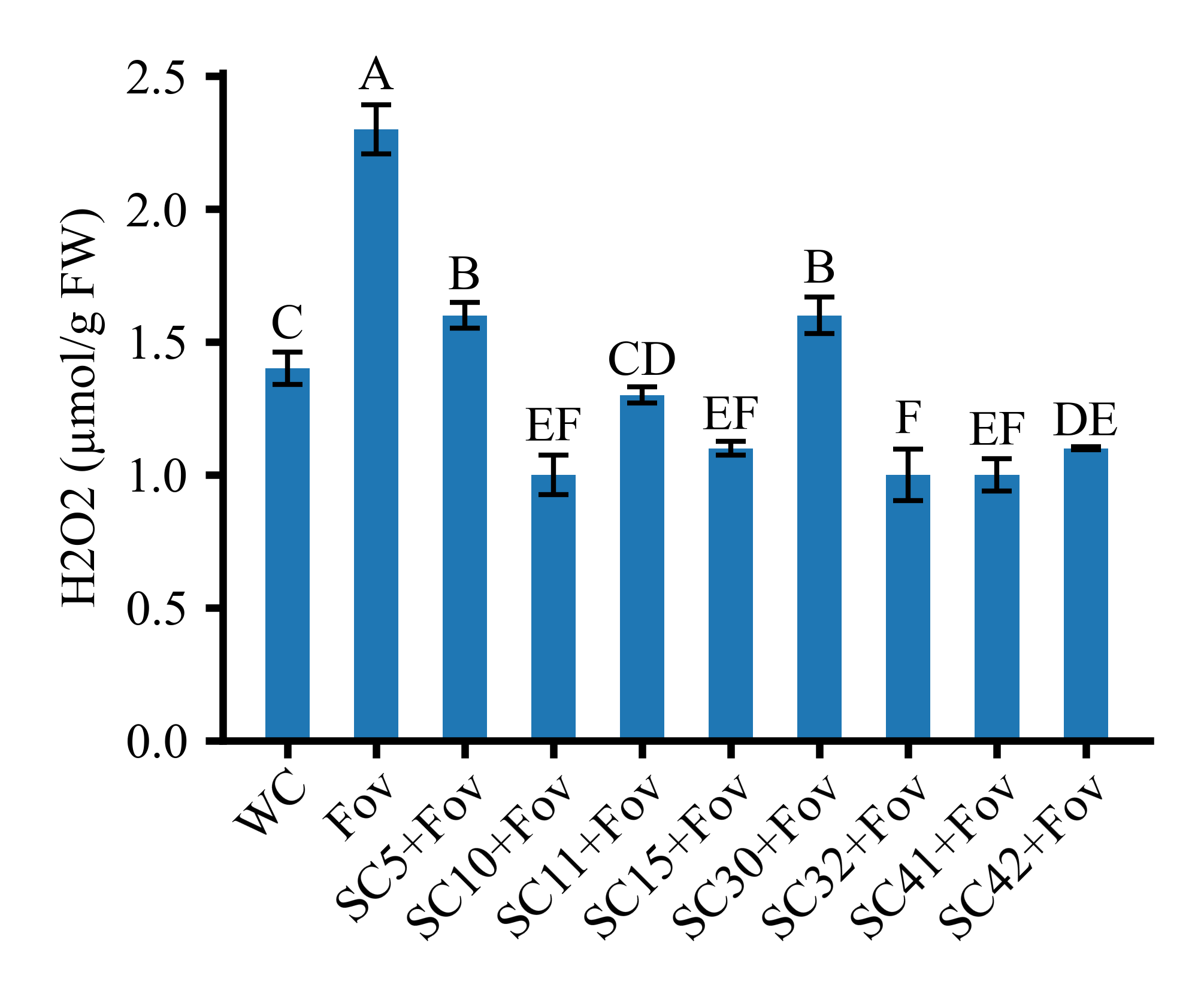

Supplement: Supplementary file 1 [file Data_Sheet_1.zip › Figure 6B.tiff]

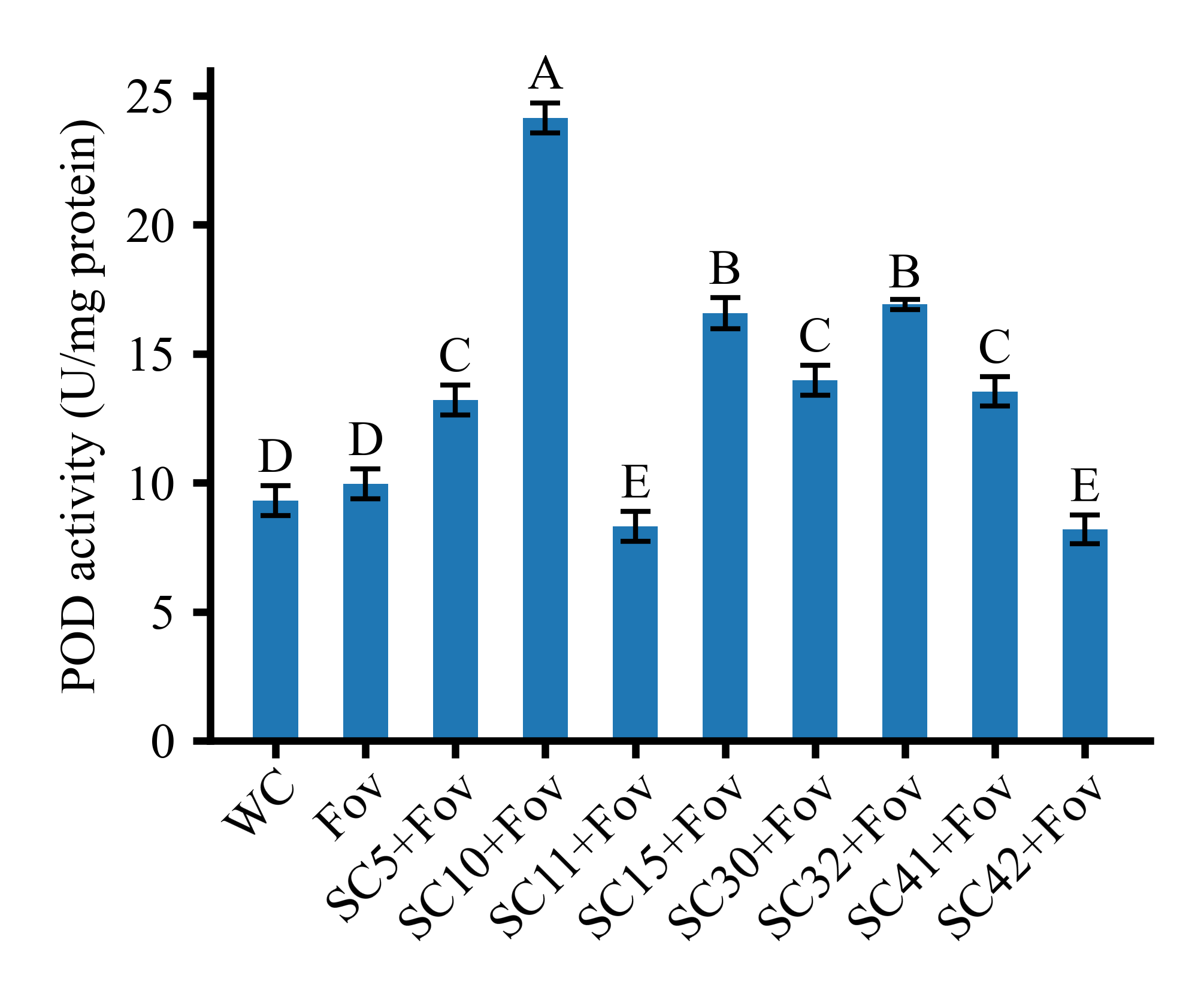

Supplement: Supplementary file 1 [file Data_Sheet_1.zip › Figure 6C.tiff]

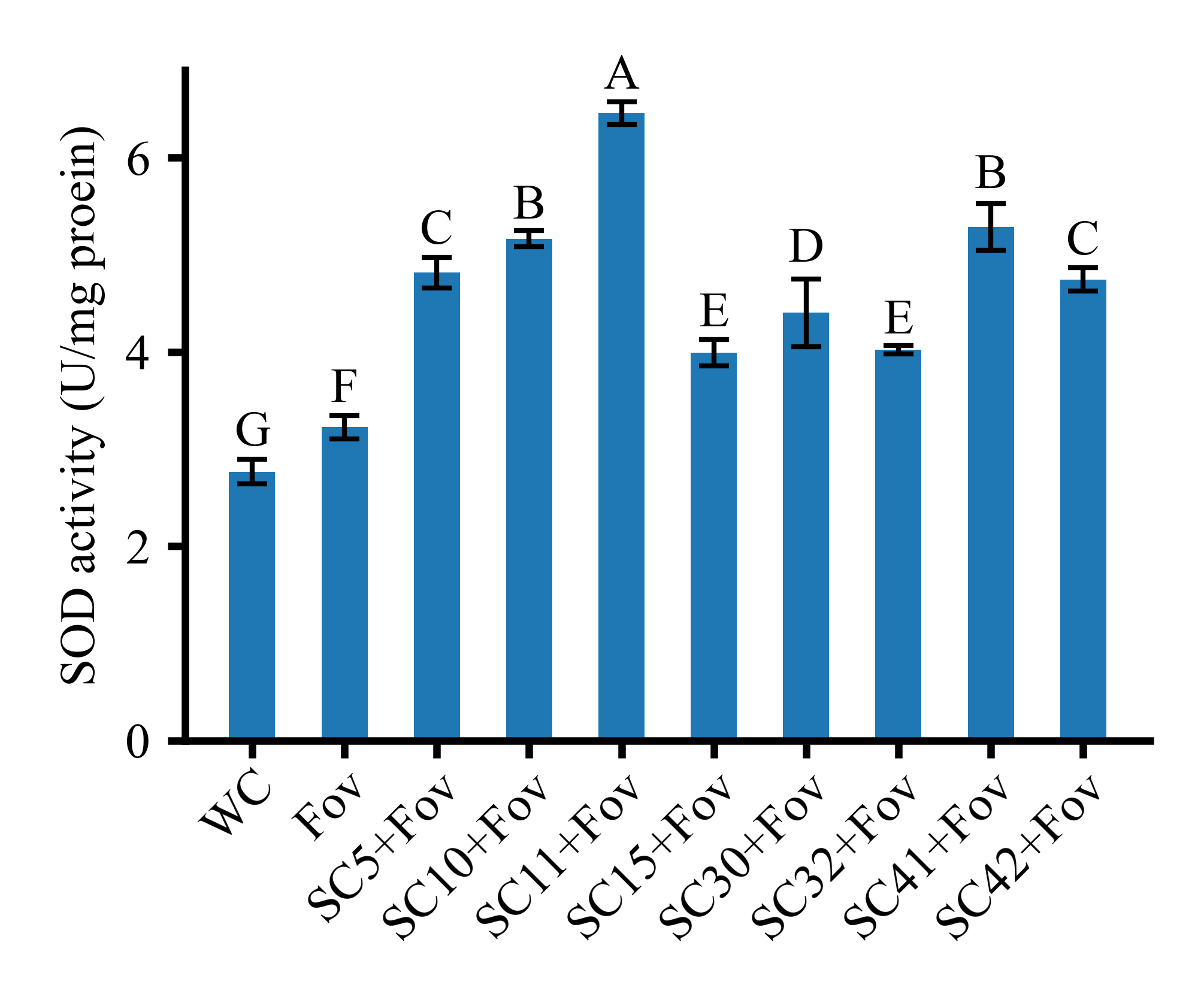

Supplement: Supplementary file 1 [file Data_Sheet_1.zip › Figure 6D.tiff]

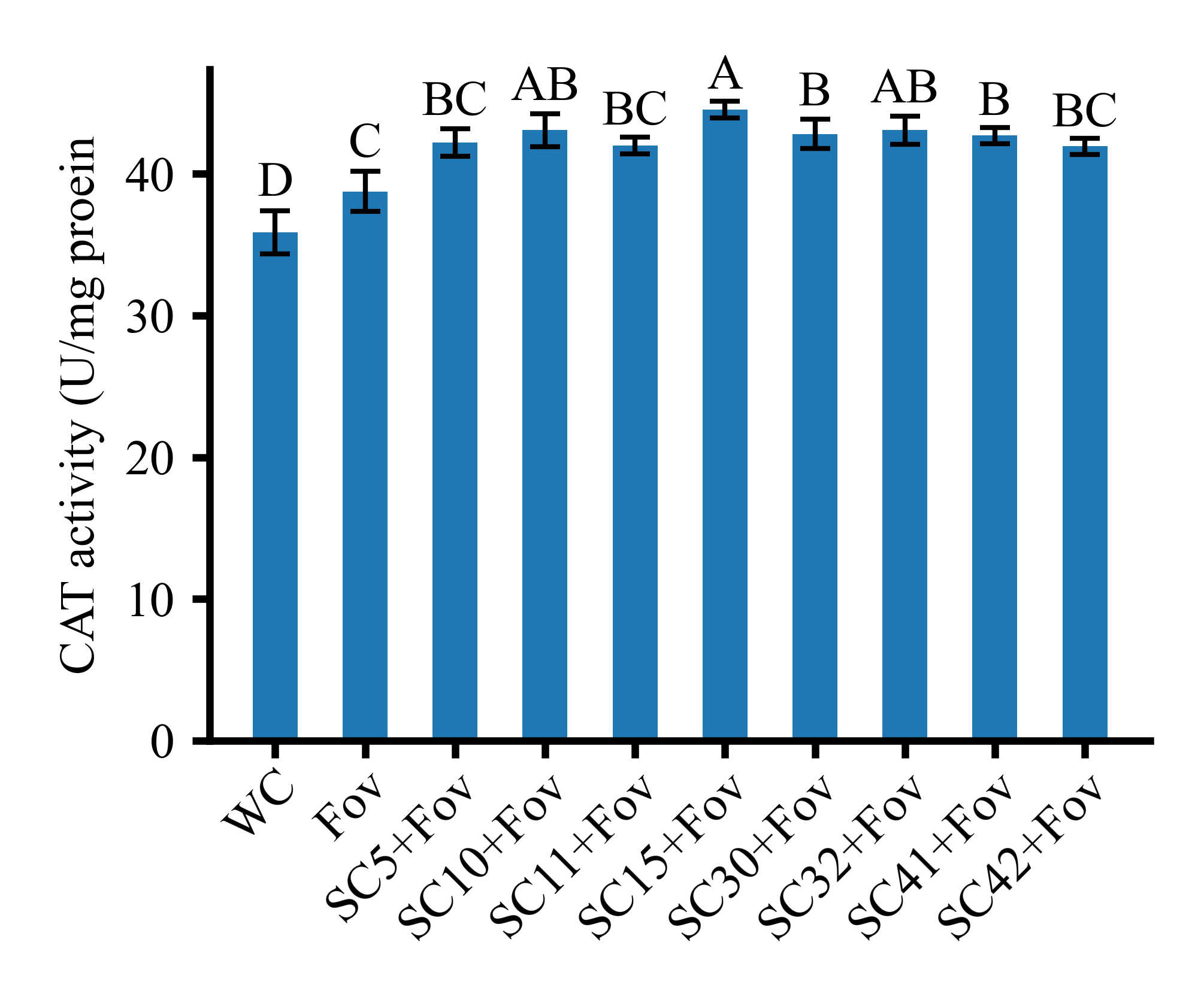

Supplement: Supplementary file 1 [file Data_Sheet_1.zip › Figure 6E.tiff]

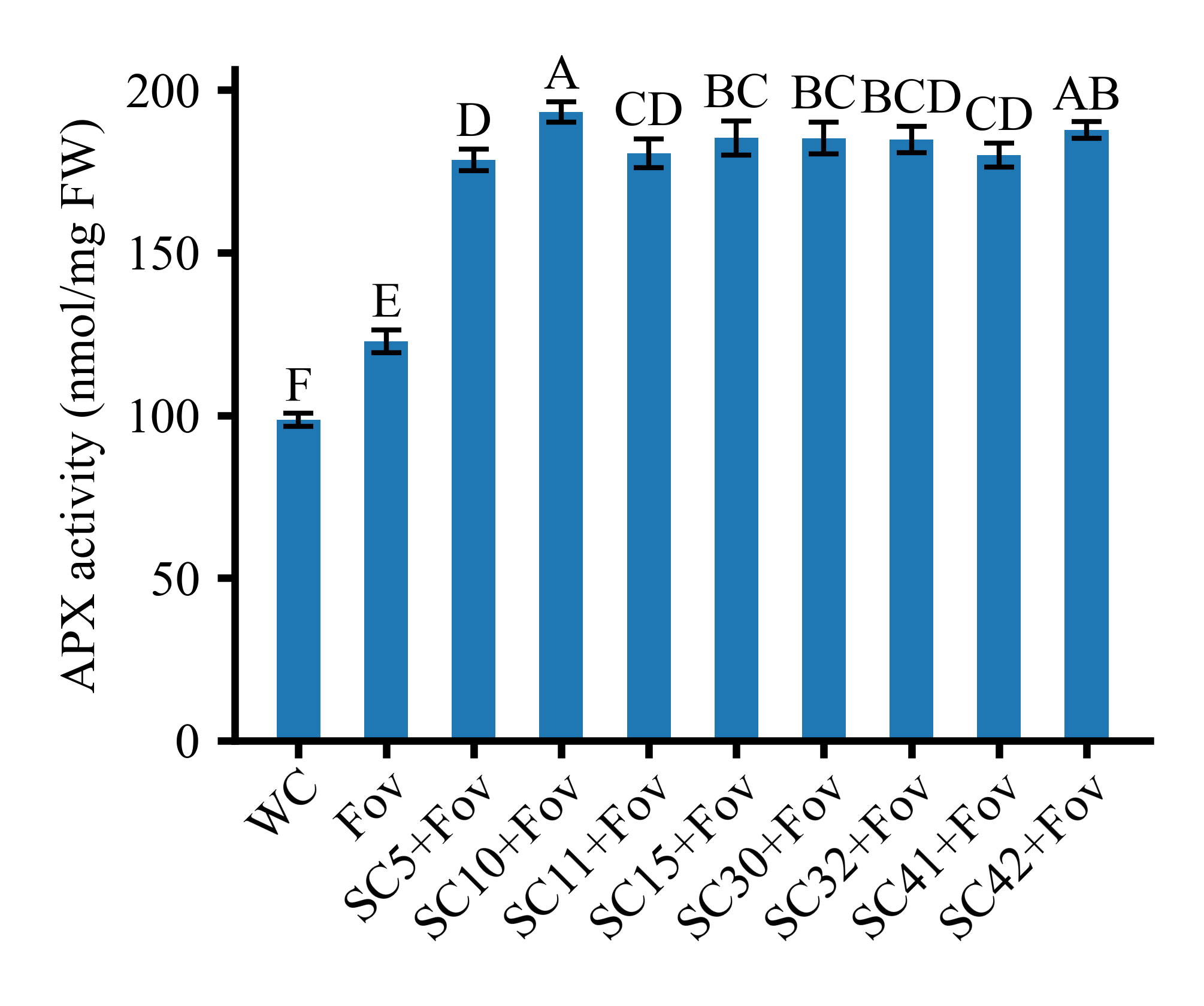

Supplement: Supplementary file 1 [file Data_Sheet_1.zip › Figure 6F.tiff]
